# Videography of pathways for enteric pathogen exposure among children in urban informal settlements in Fiji and Indonesia

**DOI:** 10.1186/s12889-026-27221-7

**Published:** 2026-04-07

**Authors:** Ruzka R. Taruc, S. Fiona Barker, Genie Fleming, Josphin Johnson, Stephen P. Luby, Ansariadi Ansariadi, Autiko Tela, Shannon Zhong, Laura H. Kwong, Karin Leder, RISE Consortium

**Affiliations:** 1 School of Public Health and Preventive Medicine, Monash University, Melbourne, VIC 3004 Australia; 2 Revitalising Informal Settlements and their Environments (RISE), Makassar, Indonesia; 3 School of Public Health and Primary Care, Fiji National University, Tamavua, Suva Fiji; 4 Division of Environmental Health Sciences, School of Public Health, University of California, Berkeley, Berkeley, CA USA; 5 School of Medicine, Stanford University, Stanford, CA USA; 6 School of Biological Sciences, Monash University, Clayton, VIC 3800 Australia; 7 Faculty of Public Health, Hasanuddin University, Makassar, Indonesia

**Keywords:** Pathogen exposure risks, Mouthing, Urban informal settlements, Video observation, Fiji, Indonesia

## Abstract

**Background:**

Children in urban informal settlements are vulnerable to enteric pathogen exposures due to inadequate availability of clean water, sanitation, and wastewater treatment. These exposures can contribute to diarrhea, malabsorption, and poor growth. Understanding how children interact with their environments – particularly through mouthing behaviors – can help identify high-risk environmental sources, exposure pathways, and opportunities for intervention. The objective of the study is to characterize mouthing behaviors among young children and assess the environmental contexts in which these behaviors occur, in order to identify potential pathogen exposure risk.

**Methods:**

Two videography campaigns were conducted involving 192 children under five years old (106 in Fiji and 86 in Indonesia), with a mean observation duration of 4.1 h per child. Mouthing behaviors were recorded along with the environmental context (location and presence of risk factors (i.e., near human or animal feces, close to animals, or interacting with environmental water)).

**Results:**

All children mouthed objects during observation. Mouthing frequencies were similar across countries: 68.6 contacts/h in Indonesia and 68.2 contacts/h in Fiji. Most frequently mouthed objects were food (25.5 in Indonesia, 16.9 in Fiji), fomites (17.4 and 24.4), and the child’s own hand (17.6 and 20.4 contacts/h). Outdoors, mouthing was common: 98% of Indonesian children and 91% of Fijian children, with frequencies of 58.9 and 50.1 contacts/h, respectively. Indonesian children spent significantly more time outdoors (26.3 vs. 8.2 min/h; *p* < 0.001). High-risk contexts were observed in 66% of children in Fiji and 93% in Indonesia, with over half mouthing objects while in these settings.

**Conclusions:**

Systematically incorporating contextual information on the settings where mouthing occurs, enables a broader understanding of children’s potential pathogen exposure risks through child-environment interactions.

**Supplementary Information:**

The online version contains supplementary material available at 10.1186/s12889-026-27221-7.

## Background

The last decade has seen a shift towards more holistic One Health and Planetary Health approaches to global health challenges. Both concepts recognize that human health, and the health of animals, plants, and the wider natural environment and systems, are closely linked and interdependent [1–4]. Accordingly, they provide highly relevant frameworks for assessing human–environmental exposure risks that contribute to disease acquisition, including fecal-oral pathogen exposure pathways for gastrointestinal infection.

More than 1 billion people live in urban informal settlements [5], which are often densely populated and lack basic services and public infrastructure such as piped water, sanitation, and effective drainage networks [6]. Children living in urban informal settlements in low- and middle-income countries (LMICs) are, therefore, exposed to a confluence of environmental, social, economic and behavioral factors, many of which impact pathogen exposure and disease risks [3]. In LMICs such as Fiji and Indonesia, diarrhea - the third leading cause of all deaths in children under five years of age globally [7] - accounts for approximately 5–6% of under-five mortality [8]. In addition to causing diarrhea, recurrent or persistent infection with enteric pathogens can cause physiological and functional change in the gastrointestinal tract, reducing nutrient absorption and potentially impairing growth and cognitive development in young children [9, 10]. Most enteric pathogens which cause diarrhea are transmitted from the environment to children through the “F-pathways” – namely fingers, food, fomites, fluids, flies, and fields [10, 11].

Various methods to assess child behaviors and the F-pathways have been used, including surveys, caregiver-reports, structured/unstructured observations, and video observations [12]. Previous exposure studies on children’s behaviors have mostly focused on mouthing or touching, quantifying frequency of contacts and the type(s) of object(s) mouthed or touched. Most early studies were performed in high-income countries (HICs) such as the US [13–15] and Australia [16], but the number of healthy life years lost per person from environmental exposures has been estimated to be 15 times higher in LMICs than in HICs [1]. Local contextual information is therefore critical when evaluating child behaviors and exposure risks. Studies using structured or video observation methods have emerged more recently from South America [17], Africa [18, 19], and Asia [20–23], focusing particularly on quantifying child behaviors, soil ingestion, and mouthing of hands, food and feces, recognizing that these activities pose particular risks in contaminated environments [18, 23].

In this study, we investigated mouthing behaviors among children living in informal settlements in Suva, Fiji and Makassar, Indonesia, as part of the Revitalising Informal Settlements and their Environments (RISE) program [24]. RISE is a randomized controlled trial delivering water-sensitive infrastructure to reduce human exposure to environmental fecal pathogen contamination and to improve both environmental health as well as human health and well-being. These settlements are contaminated with both human and animal waste due to a lack of safely managed sewage and an abundance of animals living in close contact with residents [3]. Frequent flooding is also a source of environmental contamination, and exposure risks are exacerbated by poor drainage leading to stagnant water. We aimed to carefully document the mouthing behaviors (i.e. objects mouthed) of children under 5 years old, along with the environmental context in which they occurred, including location and presence of high-risk activities or contexts that are believed to increase pathogen exposure risks from mouthing episodes. Our underpinning hypothesis is that environmental context may heighten the risk from mouthing items that are prone to contamination. Therefore, adding a contextual focus to build upon previous mouthing observational studies could provide better understanding of the conditions representing potential pathways of fecal contamination exposure in urban informal settlements.

## Methods

This study was conducted as a sub study of the RISE program, based in 24 communities, half in Suva, Fiji and half in Makassar, Indonesia [24]. Observations were conducted prior to implementation of the intervention.

### Recruitment, surveys and videography details

Videography was used to observe and quantify child mouthing behaviors in their environmental contexts. Households participating in the RISE program, with at least one child under the age of 5 years, were eligible for inclusion. Information sessions were held with representatives from each settlement, and interested, eligible households were visited to provide more detailed explanations of video observation processes. A pre-consent survey was conducted to confirm demographic details and collect information about the child’s daily routine (e.g. wake-up time, meal times, nap time, where the child usually plays), which informed the scheduling of video observation visits. Verbal consent was obtained from caregivers at the pre-consent visit, and formal written informed consent was obtained on the day of video observation.

A post-video observation survey was conducted asking caregivers whether they noticed any changes during filming. The survey also recorded observations from community fieldworkers on child mobility, food storage practice, presence of feces or animal confinement around the house, and behavior change during filming. All survey questions used in this study are provided in the Supplementary Information (Tables S1, S2).

Two videography campaigns per country were performed to collect observations in both wet and dry seasons. The first campaign in each country had a target observation period of up to 4 h (minimum duration 2 h), which was deliberately extended to up to 6 h (minimum duration 4 h) in the second campaign to capture two meal times. Filming was spread across work days (Monday to Friday). The recruitment target was 6 households per settlement in campaign one and 3 per settlement in campaign two (one child per household). Recruitment was designed for a balanced proportion of age (6 months to below 2 years and 2 to 5 years, representing different mobility phases) and sex in the observed children, with planned flexibility where the exact balance was not practical.

Trained community fieldworkers filmed children (using Canon VIXIA HF R800 or Sony HDR-CX405 HD Camcorder in Indonesia, and APEMAN A87/A79 in Fiji) to record normal daytime activities in and around children’s homes, excluding sleeping, showering/bathing, toileting, diaper changes, and breastfeeding. One fieldworker (videographer) held the camera, and a second provided support by logging times for video pauses and starts and recording notes. Videographers were instructed to keep the child’s face and two hands in view at all times and to minimize interactions with children, caregivers, and other household occupants in an effort to capture the child’s normal routine. A 2–4 m distance from the child was maintained where possible to capture the child’s surroundings, and reduce interference. Staff followed children indoors and outdoors (within settlement boundaries), and caregiver presence was required at all times.

### Video coding

**Fig. 1:**
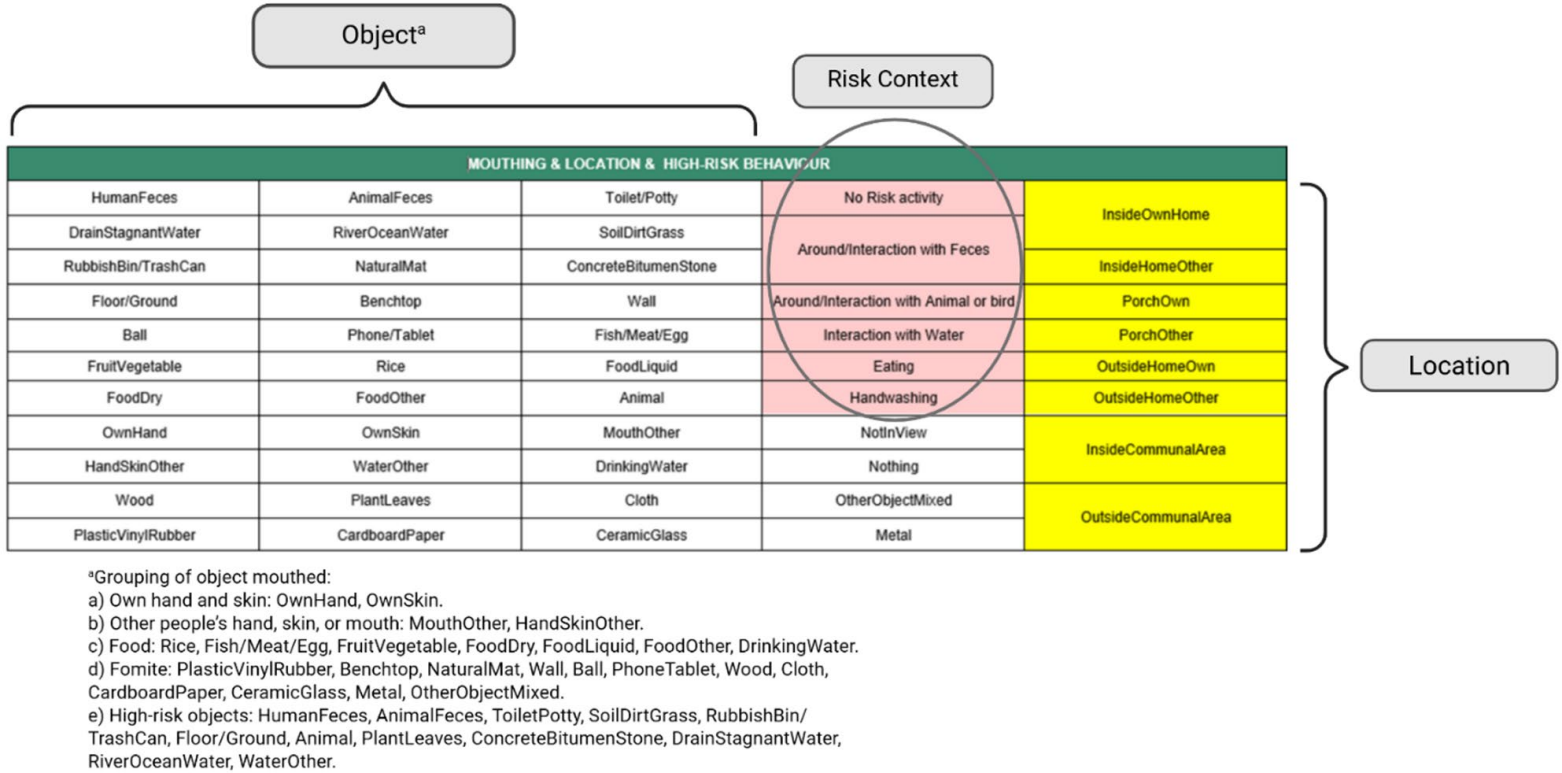
Coding palette in the LiveTrak software. Each button represents a specific object mouthed (white), a risk activity or context (pink), or a location (yellow) that reflected the child’s behavior during video recording. Object and risk contexts buttons were listed in order from high to low risk, facilitating selection of the highest risk object when more than one object or risk context was simultaneously observed

Video coding was conducted after completion of fieldwork for each campaign. Trained coders translated the video recordings into sequential text records (microlevel activity time series; MLATS) using LiveTrak software (Stanford University, CA; https://github.com/chrisdembia/LiveTrak) and a digitized coding “palette” installed on a tablet. The coding palette consisted of buttons that were grouped into three categories: objects mouthed, the child’s location (e.g., indoors/outdoors), and risk context (e.g., near feces), with a button from each category selected in parallel at all times. The palette buttons were positioned to reflect the priority risk level of the objects, from high (top left corner) to low (bottom right corner, Fig. 1). Coding focused on object mouthing, with a specific object selected whenever touched by a child’s mouth. Object categories included types of food and fomites (i.e., inanimate objects that can serve as potential intermediaries for microbial transmission to or from humans), with categories based largely on the materials objects were made of, presuming that each would have different pathogen transfer efficiencies. When mouth contact stopped the coder would select “Nothing”, or if the object being mouthed changed, the new object would be selected. If the child’s face was not visible or unclear, the coders selected “NotInView”. Only one object could be selected at a time; where multiple objects were mouthed simultaneously, the object considered to have the highest risk level was selected, prioritizing the exposure most likely to result in pathogen transfer. The pre-specified high-risk objects included: human or animal feces; toilets or potties; environmental water (e.g. stagnant water, river, or ocean water); outdoor or indoor ground surfaces (soil, dirt, grass, concrete, bitumen, floor); trash cans, plants; and animals.

The location category of the palette was used to capture both indoor and outdoor environments and to provide some inference about the distance from child’s home when outdoors. Outdoor categories were defined by area boundaries rather than specific metric distances: porch referred to the porch area of the child’s or a neighbour’s house; outside home encompassed areas outside the house structure but within the property boundary (e.g., near animal pens, front yard, or under house stilts, excluding the porch); indoor communal referred to shared indoor spaces outside the home boundary (e.g., shops, canteens, community halls, or indoor play areas); and outdoor communal referred to publicly accessible outdoor areas outside the home boundary (e.g., parks, playgrounds, creeks, or rivers). These categories therefore represent a proximal-to-distal gradient from the child’s home. The third category on the palette defined contexts conducive to exposure with three contexts reflecting higher environmental contamination: (i) being around or interacting with feces (if animal or human feces were visible in the immediate surroundings of the child); (ii) being around or directly interacting with animals; (iii) interacting with environmental water; and (iv) eating–representing possible ingestion pathway through contaminated food. Handwashing was also captured. Overall, there were 35 object categories included in the palette, eight location categories and six context categories (Fig. 1).

Video recordings were saved as multiple shorter video clips (approximately 20 min long); approximately 15% of all clips were double coded (i.e., coded separately by two people). Coders recorded timestamps and made notes whenever palette buttons were mistakenly touched or activities were missed, and LiveTrak files were edited based on these annotations to produce the final dataset. A two-way agreement single-measure intraclass correlation coefficient (ICC) was used to measure intercoder reliability, and a cut-off ICC score of ≥ 0.75 needed to be achieved during training, before coders were deemed sufficiently proficient to begin coding work. Coder assessment was conducted weekly, with ten clips double coded and ICC scores calculated for several different parameters; staff retraining was performed if ICC scores fell below 0.75. Random checks were applied to low-occurrence high-risk contexts and high-risk object mouthing. If ≥ 20% or at least 10 clips yielded an error rate ≤ 0.3, checks were stopped and errors corrected; if the error rate was > 0.3, checks continued until it fell below 0.3 or all clips were reviewed, after which all errors were corrected.

### Numerical analysis

Analyses of mouthing behaviors and their environmental context were conducted per child. Where the same child was observed in both campaigns, datasets were combined. Using the second-by-second records of children’s mouthing contacts, we calculated mouthing frequency (mouth contacts per hour observed) and the duration of time spent in each location and each risk context. Results were normalized by the total video duration (minutes per hour observed). We calculated overall mouthing frequency and frequency by grouped object types (Fig. 1). Mouthing of children’s own hand and other areas of their skin were combined as a single object group, given that the mouthing contacts predominantly involved the hand. Similarly, mouthing of other people’s hand, skin, or mouth were combined into one, as contacts with the hand and skin were most frequent within this grouping. Any food-related items were categorized as food, while all other objects or surface materials were classified as fomites. We also calculated mouthing frequency within each location or risk context and across all three categories combined to examine the intersection of mouthing with location and risk context.

While our analyses of mouthing behaviors were primarily descriptive, we conducted statistical tests where sample sizes permitted to examine systematic differences in overall mouthing frequency, location, and risk context between or within countries, based on demographic factors we hypothesized might influence these outcomes. Wilcoxon Rank Sum tests were used to examine whether, within countries, mouthing frequency (any object) or time spent outdoors differed as a function of (1) age group, (2) mobility (walking vs. pre-walking), (3) sex, or (4) location (for mouthing frequency). We also tested whether overall mouthing frequency, mouthing frequency within high-risk contexts, and time outdoors differed between countries. Chi-square tests of association with a continuity correction for 2 × 2 tables were used to evaluate within-country associations between age group or sex and the proportion of children observed in a high-risk context, where cell size allowed (< 20% of cells with counts under 5). Statistical significance was defined as *p* < 0.05. Unless indicated otherwise, observed differences among strata of interest are presented as medians or percentages. All analyses were performed using R Statistical Software v4.4.2 [25].

## Results

Videography campaigns took place from 28th February to 10th March 2022 (wet season) and 14th June to 7th July 2022 (dry season) in Fiji; corresponding dates in Indonesia were 23rd May to 8th June 2022 (dry season) and 7th to 29th November 2022 (wet season). A total of 192 children under five years old from Fiji (*n* = 106, 8 included in both campaigns) and Indonesia (*n* = 86, 23 in both campaigns) participated in video observation activities (105 boys, 87 girls), including 77 children < 2 years old and 115 children 2 to < 5 years (Table 1; see Supplementary Information, Table S3a, for more detail for each campaign). On average, children were filmed for 2.5 h in campaign 1 and 5.5 h in campaign 2, with start-times ranging from 7.20 am to 3.25 pm based on caregiver reports of usual eating patterns (Table S3a). Most children (75% in Fiji, 80% in Indonesia) were walking and not crawling (Table 1).

**Table 1:** Summary of child characteristics across the two videography campaigns combined

Child characteristics	Fiji *n* = 106	Indonesia *n* = 86	Total *n* = 192
Filming duration in hours (mean (± SD))	3.4 (± 2.1)	5.1 (± 2.7)	4.1 (± 2.5)
Age in years (mean (± SD))	2.6 (± 1.3)	2.6 (± 1.2)	2.6 (± 1.2)
Age group (number of children (%))			
6 months - <2 years old	38 (36%)	39 (45%)	77 (40%)
2 - <5 years old	68 (64%)	47 (55%)	115 (60%)
Male (number of children (%))	59 (56%)	46 (54%)	105 (55%)
Mobility (number of children (%))			
Cannot crawl	4 (4%)	1 (1%)	5 (3%)
Crawling only	13 (12%)	8 (9%)	21 (11%)
Crawling and walking	10 (9%)	8 (9%)	18 (9%)
Walking only	79 (75%)	69 (80%)	148 (77%)

### Survey reports

Most post-video survey respondents were mothers (68% in Fiji, 90% in Indonesia) or grandparents (20% in Fiji, 11% in Indonesia), nearly all of whom were grandmothers, with only one grandfather in Fiji (Table S4). The mean respondent age was 36 years, and 53% had reached at least senior high school education (Table S4). Reports of children changing their behavior in the early stage of filming (within the first hour) were more common among caregivers and fieldworkers in Fiji than Indonesia (Table S3b), mainly suggesting that children were either quieter or more talkative than usual. Caregivers in Indonesia reported that children spent more time outdoors the day before (mean = 2.9 h) and two days before (mean = 2.5 h) video observation compared with caregivers in Fiji (mean = 0.9 h and 1 h, respectively). Caregivers also reported that nearly half of the children had contact with animals (mainly cats, chickens/ducks, or domesticated birds in Indonesia; mainly dogs, cats, or chickens/ducks in Fiji; Table S4). Animal confinement structures were observed more often in Indonesian courtyards (38% vs. 5% in Fiji). Feces were observed by the fieldworkers in the house or the courtyard in 74% of houses in Fiji and 65% in Indonesia. Few caregivers (26% in Fiji, 11% in Indonesia) reported that, on the previous day, their child had mouthed soil or mud.

### Videography

#### Mouthing behaviors

All children mouthed objects during the video observation period, and overall mouthing frequency was similar between countries (68.2–68.6 contacts/h; Table 2, S5). Within countries, differences in mouthing frequency were evident in Indonesia, with girls observed to have higher rates than boys (median: 79.2 vs. 63.5 contacts/h, mean: 81.4 vs. 67.1 contacts/h, *p* = 0.017), and children who could walk exhibiting lower rates than those not yet walking (median: 65.4 vs. 96.0 contacts/h, mean: 68.9 vs. 93.5 contacts/h, *p* = 0.011) (Tables S5, S6). In contrast, mouthing frequency among Fijian children appeared consistent across age groups, sex, and mobility categories.

**Table 2:** Child mouthing by object type

Object group	Fiji (*n* = 106)				Indonesia (*n* = 86)			
	Number of children observed mouthing (%)	Mouthing frequency (contacts/h)			Number of children observed mouthing (%)	Mouthing frequency (contacts/h)		
		Mean (± SD)	Median (IQR)	Max		Mean (± SD)	Median (IQR)	Max
Any mouthing episodes	106 (100%)	71.7 (± 29.0)	68.2 (37.3)	159.4	86 (100%)	73.7 (± 30.0)	68.6 (37.9)	182.7
Food^a^	104 (98%)	18.9 (± 14.3)	16.9 (21.3)	54.9	86 (100%)	28.4 (± 14.5)	25.5 (18.7)	67.1
Fomite^b^	106 (100%)	25.6 (± 18.2)	24.4 (19.2)	129.9	86 (100%)	21.1 (± 15.9)	17.4 (17.2)	91.5
Own hand/skin^c^	106 (100%)	22.6 (± 16.1)	20.4 (15.6)	117.1	86 (100%)	19.9 (± 13.2)	17.6 (14.6)	92.8
Other people’s hand/skin^d^	95 (90%)	3.5 (± 3.6)	2.7 (5.0)	17.6	81 (94%)	3.9 (± 4.0)	2.7 (3.9)	21.4
High-risk objects^e^	58 (55%)	1.0 (± 1.6)	0.2 (1.1)	8.4	45 (52%)	0.4 (± 0.9)	0.1 (0.4)	5.5

^a^Food: rice, fish/meat/egg, fruit/vegetable, dry food, liquid food, other types of food, and drinking water

^b^Fomite: objects that are made out of plastic/vinyl/rubber, wood, cloth, cardboard/paper, ceramic/glass, metal, benchtop surface, mat from plant fiber material, wall, ball, phone/tablet, and other type of object or object with mixed materials

^c^Own hand/skin: children’s own hand and children’s skin other than hand

^d^Other people’s hand/skin: other people’s mouth and other people’s hand/skin

^e^High-risk objects: human feces, animal feces, toilet/potty, soil/dirt/grass, trash can, floor/ground, animal, plants/leaves, concrete/bitumen/stone, drainage/stagnant water, river/ocean water, and other environmental water

Across all the object categories, the most frequently mouthed object groups were fomites (observed for all children in both countries, 17.4 contacts/h in Indonesia, 24.4 contacts/h in Fiji), the child’s own hand or skin (observed for all children in both countries, 17.6 contacts/h in Indonesia, 20.4 contacts/h in Fiji), and food (observed for all children in Indonesia – 25.5 contacts/h, 98% of children in Fiji – 16.9 contacts/h; Table 2). More than half of children (52% in Indonesia, 55% in Fiji; Table 2) had at least one mouth contact with a high-risk object, but the frequency was low (median: 0.1 contact/h in Indonesia, 0.2 contact/h in Fiji; Table 2). Mouthing frequencies of each object group, stratified by age, mobility, sex, and campaign, are presented in Table S6.

#### Time outdoors

Almost all (98%) Indonesian children were observed spending time outdoors, and 83% of Fijian children went outside during the observation period (Table 3). Children in Indonesia spent significantly more of the observation period outdoors compared to children in Fiji (26.3 min/h vs. 8.2 min/h; *p* < 0.001; Table 3, S5). In Fiji, outdoor time did not differ between sexes, but was significantly different for age (3.9 min/h for < 2 years old vs. 13.5 min/h for 2 to < 5 years old; *p* = 0.012) and mobility groups (16.8 min/h of time spent outdoors among walking children vs. 0.6 min/h for others; *p* < 0.001; Tables S5, S7). No significant differences were observed in Indonesia (Tables S5, S7). In both countries, time outdoors was higher in campaign 1 (Table S7), which was during wet season in Fiji and dry season in Indonesia. In Fiji, most of the time outdoors was spent on the porch of the child’s home (55% of all time outdoors) while in Indonesia, most time was spent in a communal outdoor location (48%; Table S8); these patterns were consistent across age groups and sex (Table S8). In both countries, a greater proportion of children who were able to walk explored areas farther from their own home—such as the porch of another house, outdoor communal spaces, and in Fiji, areas outside another household—compared to those in earlier mobility stages. They also spent more time in these areas than children who were not yet walking (Table S8).

**Table 3:** Number (and proportion) of children and amount of time spent outdoors or observed in high-risk contexts

Variable	Fiji (*n* = 106)				Indonesia (*n* = 86)			
	Number of children observed (%)	Duration (min/h)			Number of children observed (%)	Duration (min/h)		
		Mean (± SD)	Median (IQR)	Max		Mean (± SD)	Median (IQR)	Max
Being outdoors	88 (83%)	17.4 (± 19.0)	8.2 (30.2)	60.0	84 (98%)	28.8 (± 18.9)	26.3 (31.9)	60.0
High-risk contexts^a^								
Any high-risk context^a^	70 (66%)	2.5 (± 6.7)	0.2 (1.9)	57.5	80 (93%)	3.5 (± 4.3)	2.2 (4.6)	19.0
Around/interaction with feces	7 (7%)	0.2 (± 1.3)	0.0 (0.0)	13.3	22 (26%)	0.1 (± 0.3)	0.0 (0.0)	2.5
Around/interaction with an animal	64 (60%)	2.0 (± 6.3)	0.1 (1.3)	57.5	79 (92%)	3.3 (± 4.2)	1.8 (4.2)	19.0
Interaction with water	25 (24%)	0.3 (± 1.4)	0.0 (0.0)	12.9	33 (38%)	0.2 (± 0.4)	0.0 (0.0)	1.7

Children may be observed in more than one high-risk context over the observation period; therefore, percentages may not total 100%

^a^High-risk contexts: Around/interaction with feces, around/interaction with an animal, interaction with environmental water

#### High-risk contexts

Most children across both countries were observed in at least one high-risk context (93% in Indonesia, 66% in Fiji; Table 3). The majority of children (92% in Indonesia, 60% in Fiji; Table 3) were observed near animals. Fewer children were seen near feces (26% in Indonesia, 7% in Fiji; Table 3) or interacting with environmental water (38% in Indonesia, 24% in Fiji; Table 3). Comparisons across age and sex within countries showed little variation in the proportion of children engaged in any of the three high-risk contexts (Table S9), but we did observe a higher proportion of children interacting with environmental water during the wet season campaign compared to the dry season (46% vs. 26%, respectively, in Indonesia; 27% vs. 13% in Fiji). Only a small proportion of time was spent engaging in high-risk contexts (≤ 2 min/h; Table 3). However, the maximum values observed were high, including one child with 58 min/h of videography spent being around animals; one child with 13 min/h being around feces, and one child with 13 min/h interacting with environmental water (Table 3).

### Intersection of mouthing behaviors, high-risk contexts, and locations

The proportion of children observed mouthing while outdoors (98% in Indonesia, 91% in Fiji; Table 4) was similar to when indoors (99% in Indonesia, 98% in Fiji; Table 4). However, mouthing frequency was significantly higher indoors than outdoors for both countries (74.7 vs. 58.9 contacts/h in Indonesia, *p* < 0.001; 71.8 vs. 50.1 contacts/h in Fiji, *p* < 0.001; Table 4, S5). This consistent pattern was largely explained by higher fomite (both countries) and food (particularly Fijian children) mouthing while indoors (Table 4), and was not influenced by age (data not shown). Eating-related mouthing (i.e., any mouthing during eating activity) accounted for 22% of total indoor mouthing in Fiji and 43% in Indonesia.

**Table 4:** Child mouthing of specific object categories by location (outdoors vs indoors) and child mouthing in high-risk contexts

Object group	Fiji (*N* = 106)								
	Outdoor (*n*=88)				Indoor (*n*=105)				*p*-value (comparison between mouthing outdoors vs indoors)
	Number of children observed mouthing (%)	Mouthing frequency (contacts/h)^a^			Number of children observed mouthing (%)	Mouthing frequency (contacts/h)^b^			
		Mean (± SD)	Median (IQR)	Max		Mean (± SD)	Median (IQR)	Max	
Any mouthing episodes	80 (91%)	54.0 (± 43.7)	50.1 (37.3)	315.8	103 (98%)	75.7 (± 34.9)	71.8 (38.1)	185.2	<0.001
Any mouthing episodes while eating	40 (46%)	10.4 (± 19.9)	0.0 (8.5)	79.5	80 (76%)	18.5 (± 20.1)	14.8 (30.9)	102.3	
Food^c^	52 (59%)	11.2 (± 18.0)	1.8 (15.7)	78.0	97 (92%)	21.3 (± 18.8)	17.1 (24.5)	92.4	
Fomite^d^	74 (84%)	17.0 (± 18.4)	11.6 (21.4)	97.1	97 (92%)	26.3 (± 20.4)	23.4 (23.1)	129.9	
Own hand/skin^e^	75 (85%)	21.0 (± 35.1)	14.1 (20.8)	315.8	100 (95%)	23.1 (± 19.0)	19.5 (18.0)	121.5	
Other people’s hand/skin^f^	45 (51%)	3.5 (± 11.2)	0.4 (3.8)	97.4	86 (82%)	3.8 (± 4.8)	1.7 (5.3)	25.0	
High-risk objects^g^	30 (34%)	1.3 (± 2.5)	0.0 (1.0)	11.3	40 (38%)	1.2 (± 5.6)	0.0 (0.5)	56.1	

Object group	Indonesia (*N* = 86)								
	Outdoor (*n*=84)				Indoor (*n*=85)				*p*-value (comparison between mouthing outdoors vs indoors)
	Number of children observed mouthing (%)	Mouthing frequency (contacts/h)^a^			Number of children observed mouthing (%)	Mouthing frequency (contacts/h)^b^			
		Mean (± SD)	Median (IQR)	Max		Mean (± SD)	Median (IQR)	Max	
Any mouthing episodes	82 (98%)	63.6 (± 38.2)	58.9 (56.4)	166.6	84 (99%)	78.9 (± 35.6)	74.7 (45.3)	193.9	<0.001
Any mouthing episodes while eating	74 (88%)	25.0 (± 21.9)	19.9 (27.2)	101.6	73 (86%)	32.0 (± 26.3)	33.0 (37.7)	106.5	
Food^c^	74 (88%)	24.1 (± 20.6)	20.0 (28.1)	86.7	77 (91%)	28.8 (± 23.0)	28.2 (32.8)	101.7	
Fomite^d^	78 (93%)	16.2 (± 14.1)	12.1 (15.9)	63.5	80 (94%)	25.6 (± 21.1)	20.8 (21.2)	102.0	
Own hand/skin^e^	81 (96%)	18.0 (± 15.2)	15.3 (14.4)	91.9	82 (97%)	21.1 (± 18.7)	15.3 (15.0)	144.9	
Other people’s hand/skin^f^	71 (85%)	4.8 (± 7.1)	2.6 (5.5)	51.3	64 (75%)	3.2 (± 4.0)	1.9 (4.0)	17.8	
High-risk objects^g^	28 (33%)	0.6 (± 2.0)	0.0 (0.3)	14.3	23 (27%)	0.3 (± 0.7)	0.0 (0.1)	3.8	

Object group	Fiji (*N*=106) Children observed in high-risk contexts^h^ (*n*=70)				Indonesia (*N*=86) Children observed in high-risk contexts^h^ (*n*=80)				
	Number of children observed mouthing (%)	Mouthing frequency (contacts/h)^i^			Number of children observed mouthing (%)		Mouthing frequency (contacts/h)^i^		
		Mean (± SD)	Median (IQR)	Max			Mean (± SD)	Median (IQR)	Max
Any mouthing episode	41 (59%)	31.6 (± 42.1)	18.6 (48.3)	215.6	69 (86%)		65.8 (± 62.3)	46.0 (67.3)	271.1

^a^Calculated by dividing mouthing counts with the child’s outdoor time, n is children who spent time outdoor

^b^Calculated by dividing mouthing counts with the child’s indoor time, n is children who spent time indoor

^c^Food: rice, fish/meat/egg, fruit/vegetable, dry food, liquid food, other types of food, and drinking water

^d^Fomite: objects that are made out of plastic/vinyl/rubber, wood, cloth, cardboard/paper, ceramic/glass, metal, benchtop surface, mat from plant fiber material, wall, ball, phone/tablet, and other type of object or object with mixed materials

^e^Own hand/skin: children’s own hand and children’s skin other than hand

^f^Other people’s hand/skin: other people’s mouth and other people’s hand/skin

^g^High-risk objects: human feces, animal feces, toilet/potty, soil/dirt/grass, trash can, floor/ground, animal, plants/leaves, concrete/bitumen/stone, drainage/stagnant water, river/ocean water, and other environmental water

^h^High-risk contexts: Around/interaction with feces, around/interaction with an animal, interaction with environmental water

^i^Calculated by dividing mouthing counts with the child’s time in high-risk contexts, n is children who spent time in high-risk contexts

Mouthing in high-risk contexts was more common among Indonesian children (86% of children observed in high-risk contexts) than among Fijian children (59%) and occurred at greater frequency (46.0 vs. 18.6 contacts/h; *p* < 0.001; Table 4, S5).

Examination of mouthing behavior across object type, location, and high-risk contexts, showed that, in both countries, mouthing frequency in non-risky contexts was higher indoors than outdoors, and the types of objects mouthed were largely consistent across locations and contexts (Fig. S1, Tables S10, S11). In Indonesia, 73% of children who mouthed objects outdoors had at least one mouthing episode in a high-risk context, compared with 24% of children who mouthed objects indoors (Fig. S1, Table S10). Among children observed in a high-risk context within each location, mouthing frequency was 45.1 contacts/h outdoors and 0.0 contacts/h (IQR: 0.0–93.0) indoors (Fig. S1, Table S11). In Fiji, 38% of children mouthing outdoors had at least one mouthing episode in a high-risk context, compared to 17% indoors (Fig. S1, Table S10). For those observed in a high-risk context, mouthing frequency was 3.4 contacts/h outdoors and 26.7 contacts/h indoors (Fig. S1, Table S11).

## Discussion

Understanding how children’s mouthing behaviors intersect with environmental contamination and exposure pathways for enteric pathogen transmission remains a central public health challenge. Assessing broad One Health risks, context-specific environmental contaminants, animal interactions, and individual child behaviors [12, 26–28] potentially helps elucidate risk exposure pathways and inform the development of preventive strategies. In this study, we report data from video observations focused on mouthing behaviors among 192 children living in urban informal settlements in Fiji and Indonesia, capturing several ‘F-pathway’ domains. By recording each mouthing event alongside its environmental context, our approach provides a unique opportunity to examine intersections between behaviors and environmental conditions, offering new insights into child exposure pathways. Children frequently mouthed objects across diverse contexts – whether spending time outdoors or in pre-defined contaminated environments – underscoring the importance of incorporating environmental context to better understand mouthing behaviors in relation to exposure risk.

All children were observed mouthing objects at least once during videography, with overall mouthing rates and most frequently mouthed object types (food, fomites, and their own hand) broadly similar across both countries. Own hand-mouthing frequencies in our cohort were lower than those reported in rural Bangladesh [23], higher than in metropolitan Taiwan [20, 29], and broadly consistent with rates observed among children in the United States [14]. Food-mouthing frequencies were comparable to those reported in Taiwan but substantially lower than those documented in Bangladesh. Non-food object mouthing frequencies in this study aligned closely with findings for Taiwanese children under 3 but exceeded those for older children, and fell within the broader ranges reported in the US [30] and Bangladesh. These findings reinforce that mouthing is a common developmental behavior in children [31], although its frequency and the types of objects mouthed vary across cultural and environmental contexts.

As an example, in Indonesia, fieldworkers observed that mealtimes for young children were not always structured around discrete breakfast and lunch periods. Instead, children frequently snacked throughout the day, often eating while engaged in play, which may in part explain higher food-mouthing rates in Indonesia, and may contribute to an increase in the likelihood of hand-to-mouth and object-to-mouth contacts. This shows how cultural feeding norms may interact with environmental contamination to shape children’s ingestion exposure.

Unlike prior studies that reported age-related declines of mouthing frequency [14, 23, 29, 30], we did not observe differences by age. Instead, in the Indonesian cohort, mobility appeared influential – likely reflecting the larger proportion of children in the walking-only stage; consistent with evidence that mobility can affect mouthing frequency [23].

Previous studies have reported child mouthing frequencies of specific high-risk objects such as soil, feces, or visibly soiled-hands/objects through video or structured observations [21–23, 32]. We grouped high-risk objects together —including soil, floor/ground surfaces, feces, and visibly dirty items— and found that over half of the children mouthed at least one such object during the observation period. Although mouthing of high-risk objects was infrequent or brief, such contacts may be sufficient for pathogen ingestion. High-risk object mouthing frequencies in our cohort were comparable to soil-mouthing reported in Bangladesh (0.4–1.3 contacts/h; [23]), albeit observed among fewer children (23%). Structured observations in other studies reported variable prevalence of soil-mouthing behaviors in children: 52% in the Democratic Republic of Congo [32], and 18% in Bangladesh [21, 22].

Building on preliminary findings of environmental contamination in urban informal settlements [3], our analysis assumed that outdoor environments pose greater risks of enteropathogen exposure. Yet few studies in Fiji and Indonesia have examined children’s outdoor time, particularly under age five [33–35]. Overall, we found that Fijian and Indonesian children spent considerably more time outdoors than reported for metropolitan Taiwan (< 10%; [20, 29]), and was closer in duration to rural Bangladesh (54%; [23]).

Consistent with our findings that Indonesian children spent significantly more time outdoors than Fijian children, fieldworker observations noted that outdoor shared community spaces in Indonesian settlements functioned as social hubs where caregivers gathered to socialise while children played together [36]. Household plots in the Fijian settlements were generally larger and more spread out than those in the Indonesian sites, and this may have contributed to communal congregation being less frequent. The more dispersed settlement layout may also partly explain why Fijian children, when outdoors, tended to remain close to their own homes [36, 37]. These differences in settlement structure and caregiving norms likely drove the observed variation in children’s outdoor behaviors, which may in turn influence their opportunities for exposure.

Outdoor play is widely recognized as beneficial [38]. We observed mouthing outdoors to be nearly as common as indoors, with rates matching or exceeding those reported elsewhere [14, 20, 23, 30], which suggests that it is important to specifically assess outdoor mouthing frequency. Capturing environmental conditions that influence these potential pathways is particularly critical, and the environmental context category incorporated into our observation tool provides a structured means to better understand these pathways. Although we specifically aimed to capture mealtimes in the second campaign – when children might be expected to mouth food, utensils, or their own hand indoors – our findings showed that less than half of indoor mouthing events were mealtime-related. This indicates that indoor mouthing is common beyond mealtimes and highlights that interventions to reduce exposure to contamination in children’s living environment should target both outdoor and indoor exposure pathways. Attention to the hygiene of indoor floor surfaces should be emphasized, given the high frequency of fomite mouthing indoors observed in this study. Further examination of indoor surface contamination would provide a better understanding of potential exposure pathways [39] and help inform targeted interventions.

Studies have examined high-risk contexts in children’s environments that may increase pathogen exposure (e.g., open drainage canals, feces, animals, solid waste) [17, 32, 40]. In our study, we pre-specified three such contexts—presence of animals, feces, or interaction with environmental water—and found that although time spent in these settings was brief, most Indonesian children and around two-thirds of Fijian children were observed in them. The most common high-risk context involved animals (dogs and cats in Fiji; cats, chickens, or ducks in Indonesia) [3]. This is particularly concerning, as the risk of enteropathogen exposures from domestic animals in LMICs is well documented [2, 41, 42] and has been associated with reduced height-for-age Z scores [32] and greater risk of diarrhea [43].

The species composition of animals observed differed markedly between countries, suggesting distinct pathogen risk profiles. While many animals can carry bacteria such *Salmonella* and *Campylobacter* spp., dogs, the most commonly observed animals in Fiji, also carry parasites such as *Giardia* and helminths [41, 42]. A recent study in an informal settlement in Kenya found that dog feces harboured the greatest diversity of pathogens, including potential human pathogens such as *Shigella* and norovirus [42]. In Indonesia, chickens and ducks were most prevalent, with poultry potentially serving as a reservoir for high-burden enteric bacterial pathogens, parasites including *Cryptosporidium*, and viruses [2, 41]. The free-roaming presence of these animals in household and play areas across both countries increases the likelihood of contamination of surfaces and objects with which children come into contact.

These interconnections – through which pathogens can spread between animals, human feces, and the environment – are more prominent in informal settlements [44]. Contact with environmental water—likely contaminated in these settings—may also represent a significant seasonal exposure pathway [3, 39]. Crucially, by linking mouthing behaviors with exposure contexts through video observations, we found that mouthing was common among children while they were in a high-risk context—more than three-quarters of Indonesian children and over half of Fijian children in this subgroup mouthed objects while in these environments, particularly outdoors. This reinforces our earlier finding that outdoor mouthing is frequent and when it occurs in contaminated contexts, the potential for pathogen exposure is even greater.

**Fig. 2:**
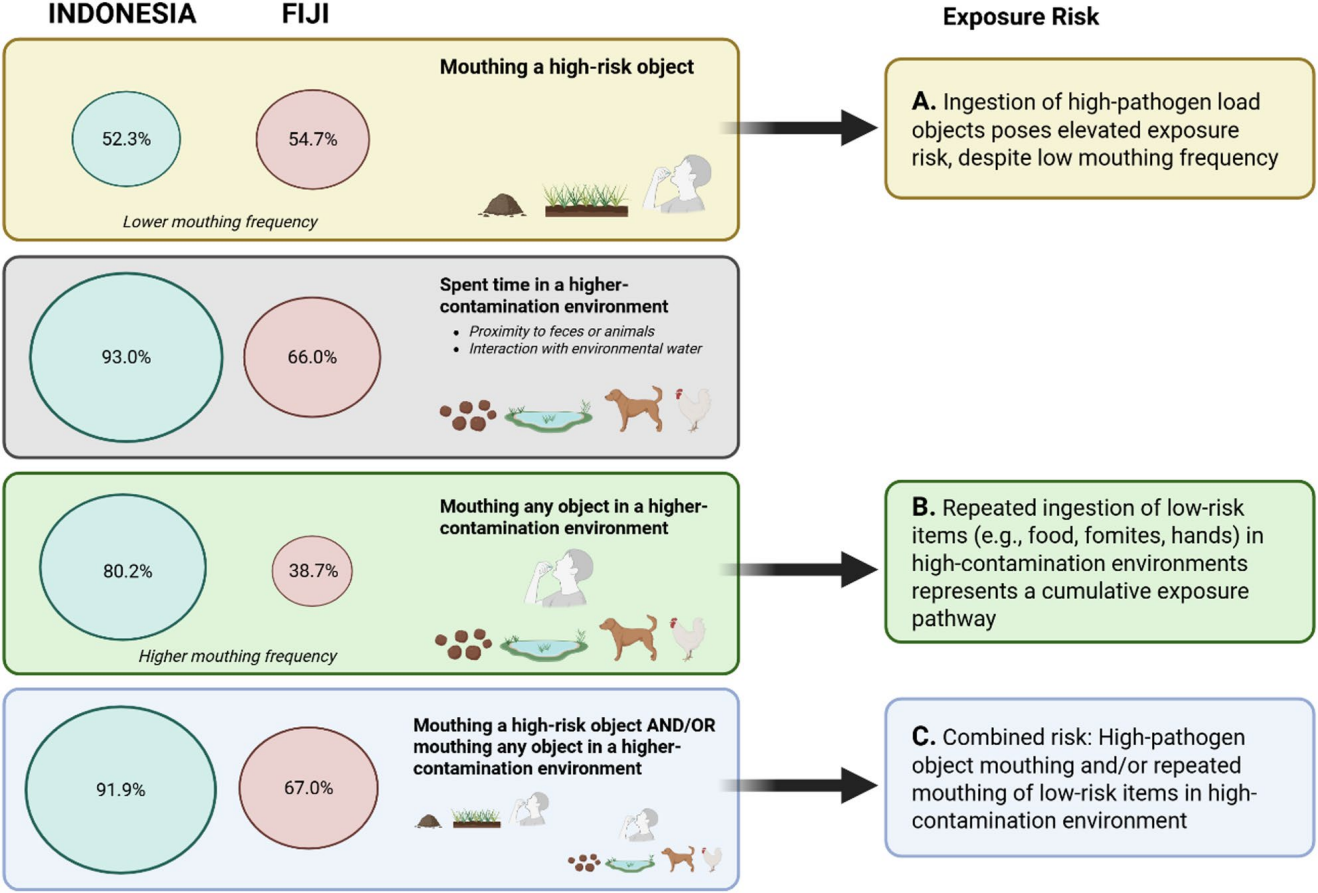
Prevalence of children in Indonesia and Fiji observed mouthing a high-risk object, mouthing any object in a higher-contamination environment, or exhibiting either or both behaviors, along with the associated exposure risk for each mouthing context

While previous research has focused on mouthing of high-risk objects (e.g., soil or feces), our findings highlight the potential importance of considering lower-risk objects (e.g., food, fomite, or children’s hand) mouthed within high-risk contexts, as well as chronic exposure to lower-risk items. Although high-risk object mouthing was comparable across countries, Indonesian children more frequently spent time in higher contamination environments (i.e., based on the predefined high-risk contexts) and engaged in mouthing behaviors there, most commonly involving food, fomites, or hands (Fig. 2). Consequently, nearly all children experienced at least one high-risk exposure factor (either mouthing a high-risk object and/or mouthing common objects in a higher-contamination environment), especially in Indonesia (Fig. 2). Although children in Fiji spent significantly less time outdoors – and mouthing in higher-contamination environments often occurred outdoors – this reduced outdoor time did not translate into proportionally lower exposure risk (Fig. 2). Our case study suggests that in addition to assessing exposure risk based on the amount of time children spend outdoors and their mouthing behaviors, the environmental context in which outdoor play occurs should also be captured and may provide a stronger approximation of risk, particularly for relevant behaviors such as hand-to-mouth or object-to-mouth contacts.

Observations on the object mouthing frequencies and prevalence gives insight into the potential F-pathways from food, fomites, finger (from hand mouthing), field/floor (from high-risk object mouthing including soil and floor) to children’s mouths. If paired with environmental sampling data of these objects, it could provide a better understanding of pathogen exposure risks. Incorporating the environmental context in which mouthing occurred further illuminates indirect F-pathways. Close proximity to animals or feces suggests potential pathogen transfer directly or from feces to fields first, then to fingers, and subsequently to mouths, either directly or through food or fomites. Children’s interaction with environmental water indicates additional opportunities for pathogen transfer. These context-specific F-pathway insights suggest that interventions should be tailored to the key transmission routes in each setting, and infers that, for example, improving domestic animal management practices and prioritizing handwashing and food hygiene promotion in environments with frequent animal contact could be important, alongside broader community-level interventions aimed at reducing environmental pathogen presence through improved water and sanitation infrastructure.

Building on this, our multi-layered results – which overlay mouthing events with environmental risk contexts – provide new insights for exposure assessment. Our synthesis emphasizes the need to examine not only the frequency of mouthing contacts, but also the location and activities associated with those events. Measurements of pathogen concentrations in the surrounding environment is also crucial to complement behavior data for risk assessment [45]. Given that a limited number of object categories—food, fomites, and hands—accounted for most mouthing events across settings, future studies may benefit from prioritizing contextual indicators of elevated exposure risk over exhaustive object-level coding. Understanding these contextual factors is particularly important in urban informal settlements, where limited access to basic services such as sanitation, clean water, drainage, and waste management can contribute to higher levels of environmental contamination. Incorporating these contextual dimensions proved valuable in our study and offers a framework for developing observation methods to inform future research in other contexts.

Seasonal conditions are known to influence children’s activity patterns and environmental exposures, yet our findings suggest that these effects may be more nuanced than expected. While Indonesian children spent descriptively more time outdoors during the dry season, consistent with the assumption that dry conditions facilitate outdoor play, this pattern was not replicated in Fiji, where outdoor time was overall less frequent and showed no corresponding seasonal trend. Despite these differences in time spent outdoor, overall mouthing frequency and high-risk object mouthing were comparable between seasons in both countries. One consistent seasonal signal observed in both countries was that children’s interaction with environmental water was higher during the wet season, reflecting greater availability of surface water and puddles during periods of rainfall.

This study has several limitations. First, we did not measure the direct impact of these exposures on child infection or health outcomes, and the exposure risks discussed were inferred from previous studies that suggest these contexts pose higher risks to child health and development. Nevertheless, baseline results from our study settlements in Indonesia showed elevated level of *E. coli* in soil, sediment, environmental water samples [3], indicating potential fecal contamination and risks when children interact with these environments. Second, we focused exclusively on mouthing and did not record hand-touching events. Previous exposure pathways models have illustrated multiple transmission routes, in which pathogens can transfer from a contamination source to the hand, and subsequently to the mouth, either directly or via food [46]. While mouthing does not directly equate to ingestion, frequent hand-to-mouth actions, particularly following surface contact, may elevate exposure risk. To better capture these pathways, future studies could incorporate behavioral sequences such as object-touching followed by hand-mouthing, especially in settings where contamination risks are high. Third, our definition of high-risk contexts was limited to presence of animals, feces, or interaction with environmental water. Other risks, such as presence of solid waste or play near a communal latrine, may also be relevant in different settings. We therefore recommend that definitions of high-risk contexts be adapted to local conditions to improve applicability across diverse environments. Fourth, we aimed to capture seasonal variation by conducting observations at two time points, including the wet season; however, wet-season observations did not consistently coincide with rainy conditions. Other climate-related factors, including elevated indoor temperatures or flooding (localized or settlement-wide), may also shape children’s activities and exposure risks but were not explored in this study. Finally, this study was conducted in an urban informal settlement setting. While the findings may not be directly generalizable to other settings or countries, the approach of capturing contextual information alongside mouthing events can be extended to other settings (e.g. rural areas or broader urban contexts), where environmental conditions and child behavioral patterns may differ.

## Conclusions

Our observations highlight the importance of considering both mouthing behavior patterns and environmental context when assessing children’s exposure risks, as indicated by the consistency of mouthing behaviors across locations and high-risk contexts. Children frequently spend time outdoors and in a variety of environmental settings, and a clearer understanding of these contexts can provide more accurate estimates of exposure risks. When defining high-risk contexts, it is important to consider not only the presence of environmental hazards but also their proximity to children, likelihood of interaction, and behavioral relevance. To better understand pathogen exposure pathways, future studies should move beyond cataloguing high-risk objects and systematically incorporate contextual information on the settings in which mouthing occurs. Objects mouthed can be categorized more broadly, focusing on distinctions between high-risk and other commonly mouthed items. Ultimately, a more nuanced understanding of child-environment interactions will be critical for developing effective strategies to mitigate pathogen exposure and improve child health outcomes in diverse settings.

## Supplementary Information

Supplementary Material 1: Figure S1. Child mouthing: intersection of objects mouthed (number and proportion of children observed and median of mouthing frequency), location, and context. Risky contexts defined as proximity to feces or animals, or interaction with environmental water. Figure displays the three most-commonly mouthed object groups that remain consistent across all intersections; full breakdown of remaining object groups is provided in Tables S10 and S11. Table S1. Pre-consent survey completed prior to the video-observation day. Table S2. Main survey completed during the video-observation day. Table S3. Child characteristics, by campaign and country (a); child behavior change during filming as reported by caregiver and fieldworker (b). Table S4. Caregiver characteristics and reports of child activities. Table S5. Wilcoxon Rank Sum test results comparing age group, mobility group, and sex within countries, and country comparison for children time outdoors; age group, mobility group, sex, and location within countries, and country comparison for all mouthing frequency; and country comparison for frequency of all mouthing during a high-risk context. Table S6. Mouthing frequency (contacts per hour), by country, age, mobility, sex, and campaign. Table S7. Time spent outdoors, by country and age, mobility, sex, and campaign. Table S8. Time spent at outdoor locations by country, sex, age and mobility. Table S9. Summary of Pearson’s Chi-squared test with Yates’ continuity correction (age and sex for each country) for proportion of children engaged in each high-risk context. Table S10. Number of children observed mouthing any object when they were outdoor vs indoor as a proportion of all children, number of children observed mouthing outdoor vs indoor in each context (as a proportion of children observed mouthing in each location), and number of children observed mouthing specific object-groups in each location-context setting (as a proportion of children observed mouthing in each location-context setting). Table S11. Median and interquartile range (IQR) of children all-objects mouthing frequency, mouthing frequency when outdoor vs indoor, mouthing frequency when outdoor vs indoor in a high-risk/other context, and mouthing frequency for each object-groups in each category of location and context.

## Data Availability

Study data will be retained for exclusive use of the study team until publication of the primary research outcomes is complete. Additional analysis that is already actively being worked on at this time will also be considered exclusive to the trial team, given their scientific contribution to the trial and the desire to allow Fijian and Indonesian scientists an opportunity to maximize their research outputs. Deidentified data will be made available at the end of the trial as approved by the ethics committees. Researchers interested in accessing data could contact the Revitalising Informal Settlements and their Environments (RISE) program with their request. Deidentified data required to reproduce this work will be available through the Monash University Research Repository (https://bridges.monash.edu). In order to uphold the RISE program’s legal and ethical obligations, including protecting confidentiality of research participants, data will be retained for exclusive use by the RISE program until the end of the randomized control trial in 2026. At the end of the research trial, deidentified data will be stored on secure Monash infrastructure and made available, upon application, as approved by relevant ethics committees. Researchers interested in accessing data may contact the RISE program (https://www.rise-program.org).
