# Supplementary material for: Videography of pathways for enteric pathogen exposure among children in urban informal settlements in Fiji and Indonesia
Source: BMC Public Health. 2026 Apr 7;26:1606. doi: 10.1186/s12889-026-27221-7 (PMC13191942; doi:10.1186/s12889-026-27221-7)
Supplement: Supplementary file 1 — Supplementary Material 1: Figure S1. Child mouthing: intersection of objects mouthed (number and proportion of children observed and median of mouthing frequency), location, and context. Risky contexts defined as proximity to feces or animals, or interaction with environmental water. Figure displays the three most-commonly mouthed object groups that remain consistent across all intersections; full breakdown of remaining object groups is provided in Tables S10 and S11. Table S1. Pre-consent survey completed prior to the video-observation day. Table S2. Main survey completed during the video-observation day. Table S3. Child characteristics, by campaign and country (a); child behavior change during filming as reported by caregiver and fieldworker (b). Table S4. Caregiver characteristics and reports of child activities. Table S5. Wilcoxon Rank Sum test results comparing age group, mobility group, and sex within countries, and country comparison for children time outdoors; age group, mobility group, sex, and location within countries, and country comparison for all mouthing frequency; and country comparison for frequency of all mouthing during a high-risk context. Table S6. Mouthing frequency (contacts per hour), by country, age, mobility, sex, and campaign. Table S7. Time spent outdoors, by country and age, mobility, sex, and campaign. Table S8. Time spent at outdoor locations by country, sex, age and mobility. Table S9. Summary of Pearson’s Chi-squared test with Yates’ continuity correction (age and sex for each country) for proportion of children engaged in each high-risk context. Table S10. Number of children observed mouthing any object when they were outdoor vs indoor as a proportion of all children, number of children observed mouthing outdoor vs indoor in each context (as a proportion of children observed mouthing in each location), and number of children observed mouthing specific object-groups in each location-context setting (as a proportion of children observ [file 12889_2026_27221_MOESM1_ESM.pdf]

Supplementary Materials

Videography of pathways for enteric pathogen exposure among children in urban informal settlements in Fiji and Indonesia

Ruzka R. Taruc[1,2], S. Fiona Barker[1], Genie Fleming[6], Josphin Johnson[1], Layla H. Kwong[4], Stephen P. Luby[5], Ansariadi[7], Autiko Tela[3], Shannon Zhong[1], Karin Leder[1] and the RISE Consortium[*]

[1] School of Public Health and Preventive Medicine, Monash University, Melbourne, VIC 3004, Australia
[2] Revitalising Informal Settlements and their Environments (RISE), Makassar, Indonesia.
[3] School of Public Health and Primary Care, Fiji National University, Tamavua, Suva, Fiji.
[4] Division of Environmental Health Sciences, School of Public Health, University of California, Berkeley; Berkeley, California, United States of America
[5] School of Medicine, Stanford University, Stanford, USA.
[6] School of Biological Sciences, Monash University, Clayton, VIC 3800, Australia
[7] Faculty of Public Health, Hasanuddin University, Makassar, Indonesia.

* Revitalising Informal Settlements and their Environments (RISE) Consortium authors, collaborators, and affiliations can be found at: doi.org/10.26180/ctjf-vf69

Figure S1. Child mouthing: intersection of objects mouthed (number and proportion of children observed and median of mouthing frequency), location, and context. Risky contexts defined as proximity to feces or animals, or interaction with environmental water. Figure displays the three most-commonly mouthed object groups that remain consistent across all intersections; full breakdown of remaining object groups is provided in Tables S10 and S11.

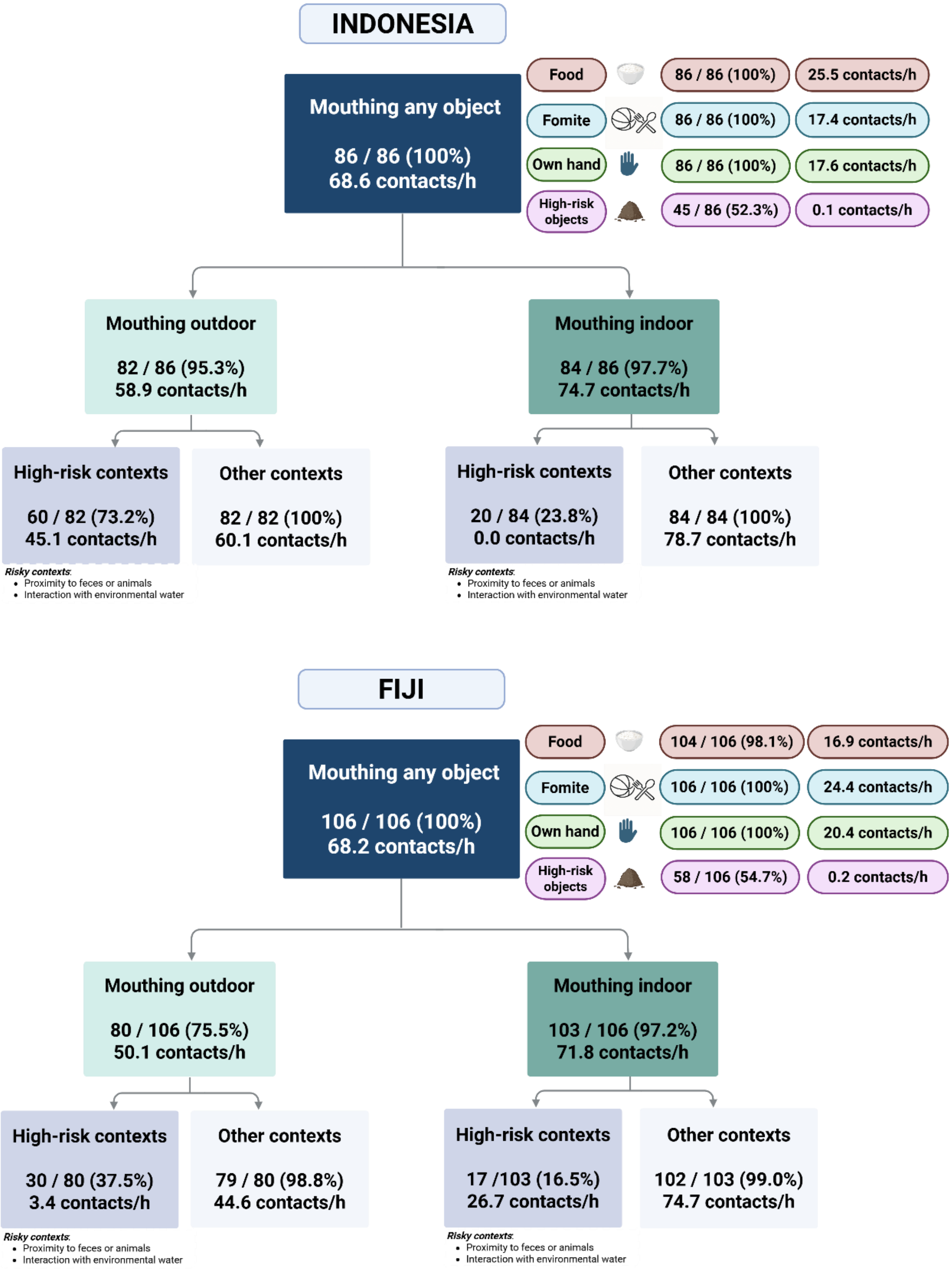

Table S1. Pre-consent survey completed prior to the video-observation day.

Survey question	Survey answer choices
B3.4: What is [child_name]'s usual wake up time?	
B3.5: What is [child_name]'s usual breakfast time?	
B3.6: What is [child_name]'s usual lunch time?	
B3.7: What is [child_name]'s usual dinner time?	
B3.8: What is/are [child_name]'s usual sleep time(s) during day? Record all the sleep times below (for example, if the child sleeps for 30 min at 10am and 2 hours at 2pm, record: "10am - 30 min; 2pm - 2 hours")	
B3.10: Where does [child_name] play/ go during the day? (select all that apply)	Indoors at home<br>Indoors at someone else's home<br>Indoors at kindergarten or school<br>Indoor communal area<br>Outdoors in own yard/close to the house<br>Outdoors under the house<br>Outdoors in someone else's yard<br>Outdoors, outside the kindergarten or school<br>Outdoors, outside the mosque (*Indonesia*) / church (*Fiji*)<br>Outdoors, near the local shops<br>Outdoors, in a community area (playground) or communal area<br>Other (specify)
B3.11: Which communal areas or to what other place does [child_name] play/ go during the day?<br>*Question relevant when: B3.10 is `Other (specify)`, `Indoor communal area`), or `Outdoors, in a community area (playground) or communal area`)*	
B3.14: Does [child_name] play inside other houses, or in the yard of other houses, in the settlement?	Yes<br>No
B3.15: Record which houses [child_name] plays in. Please record the house number. If possible, record the head of household, caregiver name or any other relevant details.<br>*Question relevant when: B3.14 is `Yes`*	

Table S2. Main survey completed during the video-observation day.

Survey question	Survey answer choices
B1.3: Record the time you start filming.	
B1.5: Did you complete a half day of videoing [child_name]?	Yes<br>No
B1.7: You have indicated that you did not complete a half day of videoing [child_name]. Please record any notes to explain why.<br>*Question relevant when: B1.5 is `No`*	
B1.8: Record what time you finished filming.	
B2.2: How are you [respondent] related to [child_name]?	Mother<br>Father<br>Aunt<br>Uncle<br>Brother<br>Sister<br>Grandmother<br>Grandfather<br>Other relative<br>Other person (not related)
CHILD TIME	
C1.1: Yesterday, what time did [child_name] wake up in the morning?	
C1.2: Yesterday, what time did [child_name] go to sleep at night?	
C1.3: Yesterday, how long did [child_name] sleep during the day? (add up all the day-time naps).<br>[Round to the nearest half-hour. If [child_name] did not sleep during the day, enter zero hours]	
C1.4: Yesterday, where did [child_name] play/ go during the day? (select all that apply)	Indoors at home<br>Indoors at someone else's home<br>Indoors at kindergarten or school<br>Indoor communal area<br>Outdoors in own yard/close to the house<br>Outdoors under the house<br>Outdoors in someone else's yard<br>Outdoors, outside the kindergarten or school<br>Outdoors, outside the mosque (*Indonesia*) / church (*Fiji*)<br>Outdoors, near the local shops<br>Outdoors, in a community area (playground) or communal area<br>Other (specify)

C1.4b: Which communal areas or to what other place did [child_name] play/ go yesterday during the day?<br>*Question relevant when: C1.4 is `Other (specify)`), `Indoor communal area`), or `Outdoors, in a community area (playground) or communal area`)*	
C1.5: Yesterday, how many hours did [child_name] spend outdoors (while awake)? (for example, total number of hours spent outdoors in the fresh air. Add up all period spent outdoors)<br>[Round to the nearest half-hour. If [child_name] did not spend any time outside, enter zero hours]	
C1.6: The day BEFORE YESTERDAY, how many hours did [child_name] spend outdoors (while awake)?<br>[Round to the nearest half-hour. If [child_name] did not spend any time outdoors, enter zero hours]	
C1.7: In the past week (7 days), on how many DAYS did [child_name] spend time outdoors?<br>[If [child_name] did not spend any time outdoors in the past week, enter zero]	
C1.8: In a typical week (7 days) during the opposite season (dry/wet season), how much time does [child_name] spend outdoors?<br>[Round to the nearest half-hour. If [child_name] does not spend any time outdoors, enter zero hours]	
C1.9: In the last week (7 days), on how many days did [child_name] leave [settlement_barcode]? (i.e. spent time outside the boundary of the settlement)<br>[If [child_name] did not spend any time outside of [settlement_barcode] in the past week, enter zero]	
FOOD	
C2.1: Yesterday, what did [child_name] eat for breakfast and/or morning snack? (select all that apply)	*Food options:*<br>Uncooked salad vegetables, unpeeled (raw)<br>Uncooked salad vegetables, peeled (raw)<br>Cooked vegetables<br>Fruit (no skin)<br>Fruit (skin)<br>Eggs<br>Poultry<br>Fish<br>Seafood (crab, shrimp, squid)<br>Shellfish (mussels, clams, etc)<br>Beef<br>Pork<br>Sausage<br>Red meat (other)<br>Lentils/dal – *in Fiji only*

	Rice<br>Fijian pastries (Topoi / Babakau / Bani lolo / Raisi valolo / waite) – *in Fiji only*<br>Traditional Makassar snack/sweets – *in Indonesia only*<br>Roti – *in Fiji only*<br>Porridge<br>Starch (rootcrops)<br>Bread<br>Biscuit / pancake / pikelets<br>Butter / margarine<br>Noodles (not instant)<br>Instant noodles<br>Leftovers from previous night<br>Chips<br>Fried food<br>Tinned food<br>Breastmilk<br>Milk (not breastmilk)<br>Formula<br>Juice<br>Tea / lemon leaf / lemon grass<br>Milo / Vico / Cocoa<br>No food in the fridge<br>Did not eat<br>Other (specify)<br>Don't know<br>Refused to answer<br>Question was not asked
C2.1b: Yesterday, what other food(s) did [child_name] eat for breakfast and/or morning snack?<br>*Question relevant when: C2.1 is `Other (specify)`*	
C2.2: Yesterday, what did [child_name] eat for lunch and/or afternoon snack? (select all that apply)	*Refer: Food options*
C2.2b: Yesterday, what other food(s) did [child_name] eat for lunch and/or afternoon snack?<br>*Question relevant when: C2.2 is `Other (specify)`*	
C2.3: Yesterday, what did [child_name] eat for dinner and/or after-dinner snack? (select all that apply)	*Refer: Food options*
C2.3b: Yesterday, what other food(s) did [child_name] eat for dinner and/or after-dinner snack?<br>*Question relevant when: C2.3 is `Other (specify)`*	
C2.4 Did you grow any of the fruit or vegetables that [child_name] ate yourself?<br>*Question relevant when: C2.1, C2.2, or C2.3 is either `uncooked salad vegetables, unpeeled (raw)`, `uncooked*	

salad vegetables, peeled (raw)`, `cooked vegetables`, `fruit (no skin)`, `fruit (skin)`	
FOOD STORAGE	
C3.1: Does the house have electricity?	Yes<br>No<br>Don't know<br>Refused to answer<br>Question was not asked
C3.2: Does the house have a refrigerator?	Yes<br>No<br>Don't know<br>Refused to answer<br>Question was not asked
C3.3: Is the refrigerator currently working and turned on (i.e. contents are cold)?<br>*Question relevant when: C3.2 is `Yes`*	Yes<br>No<br>Don't know<br>Refused to answer<br>Question was not asked
C3.4: You reported that there is a refrigerator but no electricity. Is that correct?<br>*Question relevant when: C3.1 is `No` and C3.2 is `Yes`*	Yes<br>No<br>Question was not asked
Swipe back and correct your response to the previous questions.<br>*Question relevant when: C3.4 is `No`*	
C3.5: Ask [respondent_name]: What foods do you typically keep in your fridge? (select all that apply)<br>*Question relevant when: C3.2 is `Yes`*	*Refer: Food options*
C3.5b: What other food(s) do you typically keep in your fridge?<br>*Question relevant when: C3.5 is `Other (specify)`*	
C3.8: What foods do you usually keep outside the fridge? (select all that apply)	*Refer: Food options*
C3.8b: What other food(s) do you usually keep outside the fridge?<br>*Question relevant when: C3.8 is `Other (specify)`*	
ENVIRONMENTAL EXPOSURES	
C4.1: Yesterday, how many times did you observe [child_name] putting soil, mud, clay, or sand in his/her mouth?	0 times (never)<br>1 time<br>2 times<br>3-5 times<br>More than 5 times<br>Don't know<br>Refused to answer<br>Question was not asked

C4.2: The day before yesterday, how many times did you observe [child_name] putting soil, mud, clay, or sand in his/her mouth?	0 times (never)<br>1 time<br>2 times<br>3-5 times<br>More than 5 times<br>Don't know<br>Refused to answer<br>Question was not asked
C4.3: In the past week (7 days), how many DAYS have you observed [child_name] placing soil, mud, clay or sand in their mouth during play and everyday activities? [If [child_name] did not put soil, mud, clay, or sand in his/her mouth, enter zero]	
C4.4: If you see [child_name] putting soil, mud, clay or sand in his/her mouth, what do you do? (DO NOT PROMPT)	Do nothing<br>Take the soil out of the child's mouth<br>Other (specify)<br>Don't know<br>Refused to answer<br>Question was not asked
C4.5: What other thing do you do if you see [child_name] putting soil, mud, clay or sand in his/her mouth?<br>*Question relevant when: C4.4 is `Other (specify)`*	
C4.6: If a child puts soil, mud, clay, or sand in his/her mouth and swallows, what do you think is the impact? (DO NOT PROMPT)	Eating soil is healthy<br>Eating soil is unhealthy<br>Other (specify)<br>Don't know<br>Refused to answer<br>Question was not asked
C4.7: Record what the respondent thinks is the impact if a child puts soil, mud, clay, or sand in his/her mouth and swallows<br>*Question relevant when: C4.6 is `Other (specify)`*	
C4.9: When [child_name] is outdoors - how often do they wear sandals/shoes?	Always<br>More than half the time<br>Half the time<br>Less than half the time<br>Never<br>Don't know<br>Refused to answer<br>Question was not asked
C4.10: When [child_name] is awake – how often do they wear diapers?	Always<br>More than half the time<br>Half the time<br>Less than half the time<br>Never – only wears diapers when going to sleep

	Never wears diapers (either when awake or sleeping)<br>Don't know<br>Refused to answer<br>Question was not asked
C5.1: Look at SHOWCARD 1. Can you tell me about how [child_name] interacts with these animals?	
C5.2: [child_name] plays with/touches the animal (select all that apply)	*Animal options:*<br>Dog<br>Cat<br>Chickens & ducks<br>Mongoose – *in Fiji only*<br>Domesticated birds – *in Indonesia only*<br>Wild birds<br>Bats<br>Livestock<br>Geckos<br>Rats / mice<br>Pigs – *in Fiji only*<br>Python – *in Indonesia only*<br>None<br>Other<br>Don't know<br>Refused to answer<br>Question was not asked
C5.2b: What other animal does [child_name] play with/touches?<br>*Question relevant when: C5.2 is `Other`*	
C5.3: [child_name] helps feed the animal (select all that apply)	*Refer: Animal options*
C5.3b: What other animal does [child_name] help feed?<br>*Question relevant when: C5.3 is `Other`*	
C5.4: [child_name] goes into the space where the animal sleeps (select all that apply)	*Refer: Animal options*
C5.4b: What other animal sleeping space does [child_name] go into?<br>*Question relevant when: C5.4 is `Other`*	
C5.5: [child_name] helps clean the space where the animal sleeps (select all that apply)	*Refer: Animal options*
C5.5b: What other animal sleeping space does [child_name] help clean?<br>*Question relevant when: C5.5 is `Other`*	
C5.6: When did you last replace the floor mats?	In the past week<br>In the past month<br>In the past 6 months<br>In the past year<br>More than a year ago

	Never<br>Household does not have any floor mats<br>Other<br>Don’t know<br>Refused to answer<br>Question was not asked
C5.6b: When did you last replace the floor mats?<br>*Question relevant when: C5.6 is `Other`*	
C5.7: When did you last sun the floor mats?	In the past week<br>In the past month<br>In the past 6 months<br>In the past year<br>More than a year ago<br>Never<br>Household does not have any floor mats<br>Other<br>Don’t know<br>Refused to answer<br>Question was not asked
C5.7b: When did you last sun the floor mats?<br>*Question relevant when: C5.7 is `Other`*	
CHILD AND RESPONDENT BEHAVIOUR	
C6.1: Ask [respondent_name]: Do you think [child_name] changed their behaviour during the filming today?	Yes<br>No<br>Question was not asked
C6.2: Counting from the start of the filming, how long do you think [child_name]'s altered behaviour lasted?	
C6.3: How did [child_name]'s behaviour change compared to normal?<br>*Question relevant when: C6.1 is `Yes`*	Child was quieter / more shy than usual<br>Child talked more / was louder than usual<br>Child moved around inside the house less than usual<br>Child went outside less than usual<br>Child played differently / with different objects than usual<br>Child interacted differently with animals than usual<br>Child ate more, less or different foods than usual<br>Other<br>Don’t know<br>Refused to answer<br>Question was not asked

C6.4: You reported that [child_name] played differently and/or with different objects than usual. Please explain how [child_name]'s behaviour was different.<br>*Question relevant when: C6.3 is 'Child played differently / with different objects than usual'*	
C6.5: You reported that [child_name] interacted differently with animals than usual. Please explain how [child_name]'s behaviour was different.<br>*Question relevant when: C6.3 is 'Child interacted differently with animals than usual'*	
C6.6: You reported that [child_name] ate more/less or different foods than usual. Please explain how [child_name]'s behaviour was different.<br>*Question relevant when: C6.3 is 'Child ate more, less or different foods than usual'*	
C6.7: You reported that [child_name]'s behaviour was different in another way. Please explain how [child_name]'s behaviour was different.<br>*Question relevant when: C6.3 is 'Other'*	
C7.1: Ask [respondent_name]: Do you think your interactions with [child_name] changed during the filming today?	Yes<br>No<br>Question was not asked
C7.2: Counting from the start of the filming, how long do you think your altered behaviour lasted?	
C7.3: Please explain how your interactions with [child_name] changed during the filming today.<br>*Question relevant when: C7.1 is `Yes`*	
B3.9: Is [child_name]'s weekend schedule substantially different to weekday schedule? (If the schedule is different, record how the weekend differs from the weekday schedule).	No difference between weekend and weekday<br>No kindergarten/school on the weekends<br>More time with caregivers on the weekends as they are not working<br>More time with siblings on the weekends<br>More time with friends on the weekends<br>More time with friends on the weekdays<br>More times out of the settlement on weekends<br>More times out of the settlement on weekdays<br>Goes to church / mosque on the weekends<br>More large family gatherings on the weekends<br>Other (specify)

	Don’t know<br>Refused to answer<br>Question was not asked
B3.10: Explain the other ways that [child_name]'s weekend schedule is substantially different to their weekday schedule<br>*Question relevant when: B3.9 is 'Other (specify)'*	
OBSERVATIONS BY THE FIELD WORKER	
C8.1: [Explain to [respondent_name] that you would like to walk around the house and outdoor areas to make some observations].<br>Did [respondent_name] agree to let you walk around the house?<br><br>[REMINDER: DRAW A SKETCH OF THE HOUSE AND PROPERTY AS YOU DO YOUR OBSERVATIONS]	Yes<br>No<br>Question was not asked
C8.2: Record [child_name]’s level of mobility	Cannot yet crawl at all; only sitting<br>Crawling but not yet walking at all<br>Crawling and walking<br>Walking and not crawling at all<br>Child is disabled and unable to walk<br>Not able to observe
C8.3: Observe: Do you see any raw salad, fish/seafood/shellfish/meat, or rice/porridge that is outside the fridge? (select all food items that you can see)	Raw salad<br>Fish / seafood / shellfish / meat<br>Rice / porridge<br>None of these<br>Don’t know<br>Refused to answer<br>Question was not asked
Go to the place where cooked food is stored.<br>C8.4: Is food stored covered (container has a lit or container is in a closed cupboard)?	There is no stored food<br>No, none of the food is stored with a cover<br>Most of the food is stored without a cover<br>Most of the food is stored with a cover<br>All of the food is stored covered<br>Unable to observe food storage area
C8.5: Approximately how many flies do you see in the food preparation/food storage area?	None<br>1-9<br>10-24<br>25+<br>Don’t know
Now you will observe different locations and record any feces you see. Go inside the house.	Human<br>Used diaper<br>Dog

C9.1: Which types of feces do you see? (select all that apply)	Cat<br>Chickens/Duck<br>Goat<br>Rodent (rat / mice / mongoose)<br>Pig – *for Fiji only*<br>Cow – *for Indonesia only*<br>Other animal<br>Animal source of feces unknown<br>No feces are visible
C9.1b: What other type of animal feces do you see?<br>*Question relevant when: C9.1 is 'Other animal'*	
C9.2: How many total piles of feces and/or used diapers do you see inside the house?	
Go to the courtyard / area surrounding the house.<br>C9.3: Which types of feces do you see?	Human<br>Used diaper<br>Dog<br>Cat<br>Chickens/Duck<br>Goat<br>Rodent (rat / mice / mongoose)<br>Pig – *for Fiji only*<br>Cow – *for Indonesia only*<br>Other animal<br>Animal source of feces unknown<br>No feces are visible
C9.3b: What other type of animal feces do you see?<br>*Question relevant when: C9.3 is 'Other animal'*	
C9.4: How many total piles of feces and/or used diapers do you see in the courtyard?	
C9.5: Are there any animal confinement structures? If yes, indicate what type of confinement structures there are.	Pig – *for Fiji only*<br>Cow – *for Indonesia only*<br>Ducks<br>Chickens<br>Dogs<br>Goats<br>Other animal<br>No animal confinement structures<br>Don't know
C9.6: What other animal is the confinement structure for?<br>*Question relevant when: C9.5 is 'Other animal'*	
C9.7: Go inside the pig (*for Fiji*) / cow (*for Indonesia*) confinement structure. How many total piles of feces do you see in the pig (*for Fiji*) / cow (*for Indonesia*) confinement?<br>*Question relevant when: C9.5 is 'Pig / Cow'*	

C9.8: Go inside the duck confinement structure. How many total piles of feces do you see in the duck confinement?<br>*Question relevant when: C9.5 is 'Ducks'*	
C9.9: Go inside the chicken confinement structure. How many total piles of feces do you see in the chicken confinement?<br>*Question relevant when: C9.5 is 'Chickens'*	
C9.10: Go inside the dog confinement structure. How many total piles of feces do you see in the dog confinement?<br>*Question relevant when: C9.5 is 'Dogs'*	
C9.11: Go inside the goat confinement structure. How many total piles of feces do you see in the goat confinement?<br>*Question relevant when: C9.5 is 'Goats'*	
C9.12: Go inside the [animal_confinement_other] structure. How many total piles of feces do you see in the [animal_confinement_other] confinement?<br>*Question relevant when: C9.5 is 'Other animal'*	
D2.1: While you were filming today, how much do you think [child_name] changed his/her behaviour from normal?	Not at all<br>A little<br>A fair amount<br>A great deal<br>Not relevant<br>Don't know
D2.2: While you were filming today, how much do you think non-target child/ren changed their behaviour from normal?	Not at all<br>A little<br>A fair amount<br>A great deal<br>Not relevant<br>Don't know
D2.3: While you were filming today, how much do you think adults changed their behaviour from normal?	Not at all<br>A little<br>A fair amount<br>A great deal<br>Not relevant<br>Don't know
D2.4: Counting from the start of the filming, how long do you think [child_name]'s altered behaviour lasted?	
Indonesia only:<br>D2.5: Where was the kitchen located?	*Area location options:*<br>Indoors (inside the house)<br>Outdoors (outside the house, in the porch, in an outdoor area)<br>Both - there is one indoors (inside the house) and a second one outdoors<br>This household does not have one

	Did not observe
Indonesia only:<br>D2.6: Where was the laundry located?	*Refer: Area location options*
Indonesia only:<br>D2.7: Where was the bathroom/toilet located?	*Refer: Area location options*
D3.1: What was the main language used during the filming today?	*Language options:*<br>*Fiji*:<br>No other languages<br>I Taukei (Bauan dialect)<br>I Taukei (other dialect)<br>Hindi<br>English<br>Other<br>Don't know<br><br>*Indonesia*:<br>No other languages<br>Bahasa Indonesia<br>Makassar<br>Konjo<br>Bugis<br>Toraja<br>English<br>Other<br>Don't know
D3.1b: What was the main language used during the filming today?<br>*Question relevant when: D3.1 is `Other`*	
D3.2: What other languages were used during the filming? (select all that apply)	*Refer: Language options*
D3.2b: What other language(s) were used during the filming? (record any languages not selected in the previous questions)<br>*Question relevant when: D3.2 is `Other`*	
D3.3: Record any other thoughts or reflections about the impact of filming on the day-to-day activities of the family.	
D3.4: Take a photo of sketch you have made of the interior of the house	
D3.5: Take a photo of your logsheet. It should be completed with start and end times and reason for each stop in filming.	
R1.10: Record any notes, comments or observations.	

Table S3. Child characteristics, by campaign and country (a); child behavior change during filming as reported by caregiver and fieldworker (b).

(a)

	Campaign 1			Campaign 2		
	**Fiji Wet season n = 74**	**Indonesia Dry season n = 72**	**Total n = 146**	**Fiji Dry season n = 40**	**Indonesia Wet season n = 37**	**Total n = 77**
**Child characteristics**						
**Filming duration in hours (mean (± SD))**	2.0 (± 0.8)	3.0 (± 0.6)	2.5 (± 0.8)	5.2 (± 0.7)	5.9 (± 0.6)	5.5 (± 0.7)
**Age in years (mean (± SD))**	2.9 (± 1.3)	2.5 (± 1.2)	2.7 (± 1.3)	2.0 (± 1.1)	2.2 (± 1.1)	2.1 (± 1.1)
**Age group (number of children (%)):**						
6 months - <2 years old	19 (26%)	29 (40%)	48 (33%)	22 (55%)	22 (60%)	44 (57%)
2 - <5 years old	55 (74%)	43 (60%)	98 (67%)	18 (45%)	15 (41%)	33 (43%)
**Male (number of children (%))**	45 (61%)	39 (54%)	84 (58%)	20 (50%)	17 (46%)	37 (48%)
**Child's time outdoors - caregiver reported**						
Time outdoors the day before (mean (± SD) in mins)	63.8 (± 64.1)	163.3 (± 125.7)	113.5 (± 111.3)	45.8 (± 53.9)	194.6 (± 160.1)	117.3 (± 138.7)
Time outdoor two days before (mean (± SD) in mins)	65.0 (± 56.1)	145.0 (± 115.5)	105.0 (± 99.0)	47.3 (± 50.8)	166.2 (± 156.3)	104.4 (± 128.4)
Time outdoor the day before[a] (mean (± SD) in min/h)	5.8 (± 7.1)	13.3 (± 10.5)	9.5 (± 9.7)	3.5 (± 3.8)	17.2 (± 17.8)	10.1 (± 14.3)
Time outdoor two days before[a] (mean (± SD) in min/h)	6.0 (± 6.8)	11.7 (± 9.0)	8.8 (± 8.4)	3.6 (± 3.8)	15.2 (± 18.0)	9.2 (± 13.9)
Number of days children spent time outdoors in the past week (mean (± SD) in days)	3.1 (± 2.5)	6.0 (± 2.2)	3.9 (± 9.1)	2.4 (± 2.1)	5.6 (± 2.4)	3.9 (± 2.8)

[a] *Estimated time outdoor based on the proportion of reported time outdoor (min) / (reported awake time – reported nap time)*

(b)

	**Fiji n = 106**	**Indonesia n = 86**	**Total n =192**
**Behavior change during filming - caregiver reported**			
Number of children (%)	54 (51%)	22 (26%)	76 (40%)
Duration of behavior change in minutes (mean (± SD))	51.7 (± 47.1)	35.5 (± 27.2)	47.0 (± 42.8)
**Behavior change during filming - fieldworker reported**			
Number of children (%)	71 (67%)	40 (47%)	111 (58%)
Duration of behavior change in minutes (mean (± SD))	40.1 (± 26.8)	32.3 (± 14.2)	37.3 (± 23.3)
Degree of reactivity observed (number of children (%)):			
Not at all	35 (33%)	46 (54%)	81 (42%)
A little	55 (52%)	38 (44%)	93 (48%)
A fair amount	13 (12%)	2 (2%)	15 (8%)
A great deal	3 (3%)	0 (0%)	3 (2%)

Table S4. Caregiver characteristics and reports of child activities

	Fiji n = 106	Indonesia n = 86	Total n =192
**Caregiver characteristics**			
**Caregiver age in years (mean (± SD))**	37.1 (± 11.3)	34.3 (± 9.8)	35.8 (± 10.7)
**Caregiver relationship to child (number of caregivers (%))**			
Mother	72 (68%)	77 (90%)	149 (78%)
Father	7 (7%)	0 (0%)	7 (4%)
Grandparent	21 (20%)	9 (11%)	30 (16%)
Other relatives	6 (6%)	0 (0%)	6 (3%)
**Caregiver highest level of education (number of caregivers (%))**			
Never attended school	1 (1%)	2 (2%)	3 (2%)
Primary / junior high school	5 (5%)	41 (48%)	46 (24%)
Senior high school	47 (44%)	33 (38%)	80 (42%)
Academy / university	17 (16%)	5 (6%)	22 (12%)
Data not available	36 (34%)	5 (6%)	41 (21%)
**Caregiver reports of child activities**			
**Caregiver reported child time outdoor**			
Child time outdoor the day before (mean (± SD) in mins)	54.8 (± 60.4)	172.3 (± 138.0)	108.0 (± 118.3)
Child time outdoor two days before (mean (± SD) in mins)	56.8 (± 54.0)	147.6 (± 127.6)	97.9 (± 104.7)
Child time outdoor the day before[a] (mean (± SD) in min/h)	4.8 (± 6.2)	14.7 (± 14.1)	9.3 (± 11.6)
Children time outdoor two days before[a] (mean (± SD) in min/h)	5.0 (± 6.0)	12.7 (± 13.3)	8.5 (± 10.7)
**Caregiver reported the child plays with/touches animal(s)[b] (number of children (%))**	48 (45%)	42 (49%)	90 (47%)
Dog	32 (30%)	2 (2%)	34 (18%)
Cat	30 (28%)	34 (40%)	64 (33%)
Chickens & ducks	2 (2%)	17 (20%)	19 (10%)
Domesticated birds	0 (0%)	6 (7%)	6 (3%)
Other / do not know	4 (4%)	3 (4%)	7 (4%)
**Caregiver report of the child putting soil, mud, or clay in their mouth the day before observation (number of children (%))**			
Never	78 (74%)	77 (90%)	155 (81%)
1 - 2 times	13 (12%)	7 (8%)	20 (10%)
3 – 5 times	5 (5%)	1 (1%)	6 (3%)
More than 5 times	0 (0%)	1 (1%)	1 (1%)
Do not know	10 (9%)	0 (0%)	10 (5%)

[a] *Estimated time outdoor based on the proportion of reported time outdoor (min) / (reported awake time – reported nap time)*

[b] *More than one response could be selected, results for this question will not sum to 100%. No caregiver reported contact with wild birds, bats, livestock, rodents, or phyton snakes.*

Table S5. Wilcoxon Rank Sum test results comparing age group, mobility group, and sex within countries, and country comparison for children time outdoors; age group, mobility group, sex, and location within countries, and country comparison for all mouthing frequency; and country comparison for frequency of all mouthing during a high-risk context.

Variable	Combined				Indonesia			Fiji		
	n	W	P-value	Factors	n	W	P-value	n	W	P-value
**Time outdoors (min/h)**	192	6245	<0.001*	Age	86	816	0.386	106	1671	0.012*
				Mobility		527	0.522		556	<0.001*
				Sex		1009	0.444		1297	0.568
**All mouthing frequency (contacts/h)**	192	4723	0.668	Age	86	802	0.325	106	1088	0.180
				Mobility		822	0.011*		1240	0.210
				Sex		1195	0.017*		1411	0.879
				Location (indoor vs outdoor)		1011	<0.001*		902	<0.001*
**All mouthing during a high-risk context[a] (contacts/h)**	150	1746	<0.001*							

*P-value is <0.05 indicating significant difference between groups

[a]High-risk contexts: Around/interaction with feces, around/interaction with an animal, interaction with environmental water.

Table S6. Mouthing frequency (contacts per hour), by country, age, mobility, sex, and campaign.

1) Age

Object group	Fiji (n = 106) (contacts/h)		Indonesia (n = 86) (contacts/h)	
	<2 years old n = 38	2 - <5 years old n = 68	<2 years old n = 39	2 - <5 years old n = 47
**Any mouthing episode**				
Mean (SD)	76.5 (± 27.9)	69.0 (± 29.4)	78.8 (± 32.3)	69.5 (± 27.5)
Median (IQR)	75.2 (32.8)	66.5 (38.4)	70.4 (38.6)	67.4 (46.2)
Max	157.6	159.4	182.7	125.9
**Own hand / own skin**				
Mean (SD)	17.0 (± 9.3)	25.8 (± 18.2)	18.0 (± 11.1)	21.5 (± 14.7)
Median (IQR)	15.1 (12.7)	22.8 (16.5)	15.7 (13.7)	19.4 (13.7)
Max	47.2	117.1	47.3	92.8
**Other people's mouth / hand / skin**				
Mean (SD)	4.9 (± 2.9)	2.7 (± 3.7)	6.2 (± 4.7)	2.0 (± 2.1)
Median (IQR)	4.9 (4.3)	1.1 (2.7)	4.6 (5.5)	1.2 (2.7)
Max	10.8	17.6	21.4	9.0
**Food**				
Mean (SD)	18.8 (± 13.7)	19.0 (± 14.7)	30.0 (± 12.8)	27.0 (± 15.8)
Median (IQR)	17.4 (15.1)	16.5 (23.7)	28.8 (14.4)	24.2 (23.9)
Max	54.9	54.3	67.1	57.3
**Fomite**				
Mean (SD)	35.0 (± 22.0)	20.4 (± 13.2)	24.2 (± 19.5)	18.5 (± 11.8)
Median (IQR)	32.2 (18.1)	19.1 (17.5)	17.7 (17.0)	16.2 (14.8)
Max	129.9	65.6	91.5	54.3
**High-risk objects**				
Mean (SD)	0.8 (± 1.1)	1.1 (± 1.9)	0.4 (± 0.9)	0.4 (± 0.9)
Median (IQR)	0.4 (0.9)	0.0 (1.1)	0.0 (0.3)	0.2 (0.4)
Max	4.0	8.4	4.5	5.5

2) Mobility

Object group	Fiji (n = 106) (contacts/h)		Indonesia (n = 86) (contacts/h)	
	Walking* n = 79	Other n = 27	Walking* n = 69	Other n = 17
**Any mouthing episode**				
Mean (SD)	69.9 (± 30.3)	76.7 (± 24.6)	68.9 (± 25.9)	93.5 (± 37.3)
Median (IQR)	66.9 (36.5)	72.1 (30.0)	65.4 (29.2)	96.0 (41.6)
Max	159.4	157.6	132.3	182.7
**Own hand / own skin**				
Mean (SD)	25.1 (± 17.6)	15.3 (± 6.7)	19.7 (± 13.7)	20.9 (± 11.6)
Median (IQR)	22.1 (16.4)	14.9 (11.0)	17.4 (13.7)	19.1 (16.2)
Max	117.1	29.0	92.8	44.6
**Other people's mouth / hand / skin**				
Mean (SD)	2.8 (± 3.5)	5.5 (± 3.0)	2.9 (± 3.1)	7.9 (± 4.9)
Median (IQR)	1.4 (2.8)	5.8 (5.0)	2.0 (3.5)	6.3 (4.5)
Max	17.6	10.8	18.0	21.4

**Food**				
Mean (SD)	19.5 (± 14.8)	17.3 (± 13.0)	28.3 (± 15.0)	28.7 (± 12.7)
Median (IQR)	16.5 (20.3)	17.9 (19.7)	25.3 (24.2)	28.8 (14.4)
Max	54.9	46.4	67.1	66.3
**Fomite**				
Mean (SD)	21.5 (± 13.7)	37.8 (± 23.8)	17.6 (± 10.6)	35.4 (± 24.4)
Median (IQR)	19.6 (17.8)	32.7 (16.2)	15.6 (14.4)	29.9 (33.5)
Max	65.6	129.9	54.3	91.5
**High-risk objects**				
Mean (SD)	1.1 (± 1.8)	0.7 (± 1.0)	0.3 (± 0.7)	0.7 (± 1.3)
Median (IQR)	0.2 (1.1)	0.4 (0.8)	0.1 (0.4)	0.0 (0.6)
Max	8.4	4.0	5.5	4.5

*Walking refers to children who were only walking, not those who were walking and also crawling

3) Sex

Object group	Fiji (n = 106) (contacts/h)		Indonesia (n = 86) (contacts/h)	
	Female n = 47	Male n = 59	Female n = 40	Male n = 46
**Any mouthing episode**				
Mean (SD)	70.1 (± 24.3)	72.9 (± 32.3)	81.4 (± 28.3)	67.1 (± 30.1)
Median (IQR)	71.5 (35.8)	65.7 (37.8)	79.2 (47.2)	63.5 (35.0)
Max	131.9	159.4	133.7	182.7
**Own hand / own skin**				
Mean (SD)	20.1 (± 13.6)	24.6 (± 17.8)	21.6 (± 11.8)	18.5 (± 14.3)
Median (IQR)	16.4 (13.5)	21.6 (17.7)	20.6 (18.3)	15.1 (11.8)
Max	79.2	117.1	52.4	92.8
**Other people's mouth / hand / skin**				
Mean (SD)	3.1 (± 2.9)	3.8 (± 4.0)	4.1 (± 3.7)	3.7 (± 4.4)
Median (IQR)	2.6 (4.1)	2.8 (5.3)	3.7 (5.2)	2.0 (3.6)
Max	10.8	17.6	13.9	21.4
**Food**				
Mean (SD)	19.7 (± 13.6)	18.3 (± 14.9)	28.5 (± 15.7)	28.3 (± 13.6)
Median (IQR)	17.0 (17.2)	15.7 (21.7)	25.7 (21.5)	25.5 (14.2)
Max	54.3	54.9	67.1	57.3
**Fomite**				
Mean (SD)	26.5 (± 16.8)	24.9 (± 19.4)	26.9 (± 15.5)	16.1 (± 14.7)
Median (IQR)	25.0 (21.9)	22.7 (18.0)	23.0 (19.9)	12.5 (10.3)
Max	67.3	129.9	71.0	91.5
**High-risk objects**				
Mean (SD)	0.7 (± 1.2)	1.2 (± 1.9)	0.3 (± 0.6)	0.5 (± 1.1)
Median (IQR)	0.0 (0.7)	0.4 (1.7)	0.0 (0.2)	0.2 (0.5)
Max	5.1	8.4	2.7	5.5

4) Campaign

Object group	Campaign 1		Campaign 2	
	**Fiji Wet season n = 74**	**Indonesia Dry season n = 72**	**Fiji Dry season n = 40**	**Indonesia Wet season n = 37**
**Any mouthing episode**				
Mean (SD)	75.6 (± 33.6)	76.2 (± 34.7)	65.9 (± 19.6)	68.4 (± 26.3)
Median (IQR)	69.5 (43.9)	72.7 (41.9)	66.2 (26.9)	61.8 (38.5)
Max	159.4	182.7	111.7	126.9
**Own hand / own skin**				
Mean (SD)	26.2 (± 18.2)	22.7 (± 17.0)	16.6 (± 7.8)	14.6 (± 8.0)
Median (IQR)	23.6 (19.9)	19.7 (18.4)	15.1 (10.9)	12.4 (13.8)
Max	117.1	92.8	39.6	34.0
**Other people's mouth / hand / skin**				
Mean (SD)	3.3 (± 3.6)	4.7 (± 5.6)	3.7 (± 3.4)	3.6 (± 3.0)
Median (IQR)	2.6 (3.8)	2.5 (5.1)	2.2 (5.8)	2.4 (4.3)
Max	17.6	25.3	10.8	10.3
**Food**				
Mean (SD)	18.4 (± 19.6)	26.4 (± 16.2)	22.9 (± 9.0)	30.4 (± 14.6)
Median (IQR)	12.6 (22.6)	23.4 (20.3)	22.7 (10.7)	25.7 (16.2)
Max	106.7	67.1	51.5	74.9
**Fomite**				
Mean (SD)	26.5 (± 19.2)	22.0 (± 19.1)	22.2 (± 15.9)	19.4 (± 13.6)
Median (IQR)	25.9 (19.2)	17.4 (13.4)	19.5 (15.3)	15.7 (16.9)
Max	129.9	105.2	69.7	59.9
**High-risk objects**				
Mean (SD)	1.2 (± 1.9)	0.4 (± 0.9)	0.6 (± 0.9)	0.3 (± 0.6)
Median (IQR)	0.0 (1.3)	0.0 (0.4)	0.2 (0.6)	0.0 (0.3)
Max	8.4	5.5	3.2	2.7

Table S7. Time spent outdoors, by country and age, mobility, sex, and campaign.

**Being outdoors (min/h)**	**Fiji (n = 106)**		**Indonesia (n = 86)**	
**Age groups**	**<2 years old n = 38**	**2-<5 years old n = 68**	**<2 years old n = 39**	**2-<5 years old n = 47**
Mean (SD)	12.9 (± 17.9)	20.0 (± 19.2)	30.8 (± 19.7)	27.2 (± 18.3)
Median (IQR)	3.9 (20.9)	13.5 (29.5)	29.5 (36.7)	25.8 (24.9)
Max	59.8	60.0	60.0	59.6
**Mobility**	**Walking* n = 79**	**Other n = 27**	**Walking* n = 69**	**Other n = 17**
Mean (SD)	20.5 (± 18.9)	8.4 (± 16.3)	29.6 (± 19.7)	25.7 (± 15.4)
Median (IQR)	16.8 (30.2)	0.6 (7.5)	26.5 (36.5)	22.5 (26.2)
Max	60.0	59.8	60.0	50.7
**Sex**	**Female n = 47**	**Male n = 59**	**Female n = 40**	**Male n = 46**
Mean (SD)	17.0 (± 20.3)	17.7 (± 18.0)	29.9 (± 17.9)	27.9 (± 19.9)
Median (IQR)	6.8 (30.7)	12.1 (28.8)	28.4 (24.3)	22.9 (35.5)
Max	60.0	58.7	60.0	59.6
	**Campaign 1**		**Campaign 2**	
**Campaign**	**Fiji Wet season n = 74**	**Indonesia Dry season n = 72**	**Fiji Dry season n = 40**	**Indonesia Wet season n = 37**
Mean (SD)	19.3 (± 18.9)	30.5 (± 20.3)	14.0 (± 19.0)	25.4 (± 16.9)
Median (IQR)	13.8 (29.2)	29.7 (36.8)	4.4 (23.6)	20.6 (26.2)
Max	60.0	60.0	59.8	59.9

*Walking refers to children who were only walking, not those who were walking and also crawling

Table S8. Time spent at outdoor locations by country, sex, age and mobility.

1) By country

	**Fiji (n = 88)**	**Indonesia (n = 84)**
	**Number of children (%)**	**Number of children (%)**
Porch of own home	71 (81%)	69 (82%)
Porch of another house	19 (22%)	55 (66%)
Outside own home	61 (69%)	57 (68%)
Outside another house	38 (43%)	60 (71%)
Outside in communal area	40 (46%)	77 (92%)
	**Proportion of time outdoors in % Mean (± SD)**	**Proportion of time outdoors in % Mean (± SD)**
Porch of own home	54.8 (± 43.6)	25.7 (± 31.6)
Porch of another house	4.5 (± 17.0)	9.0 (± 15.9)
Outside own home	20.3 (± 30.7)	11.2 (± 19.6)
Outside another house	7.4 (± 16.8)	6.2 (± 12.2)
Outside in communal area	13.1 (± 23.3)	47.8 (± 33.7)

2) By age

	**Fiji**		**Indonesia**	
	<2 years old n = 26	2 - <5 years old n = 62	<2 years old n = 38	2 - <5 years old n = 46
	**Number of children (%)**		**Number of children (%)**	
Porch of own home	20 (77%)	51 (82%)	30 (79%)	39 (85%)
Porch of another house	4 (15%)	15 (24%)	25 (66%)	30 (65%)
Outside own home	17 (65%)	44 (71%)	26 (68%)	31 (67%)
Outside another house	8 (31%)	30 (48%)	30 (79%)	30 (65%)
Outside in communal area	11 (42%)	29 (47%)	34 (90%)	43 (94%)
	**Proportion of time in % Mean (± SD)**		**Proportion of time in % Mean (± SD)**	
Porch of own home	52.8 (± 47.0)	55.6 (± 42.5)	26.4 (± 33.9)	25.0 (± 29.9)
Porch of another house	7.2 (± 25.0)	3.4 (± 12.3)	10.7 (± 18.6)	7.7 (± 13.2)
Outside own home	21.7 (± 34.5)	19.6 (± 29.3)	10.2 (± 21.7)	12.1 (± 18.0)
Outside another house	6.3 (± 19.1)	7.8 (± 15.8)	8.7 (± 15.5)	4.2 (± 8.2)
Outside in communal area	12.1 (± 20.8)	13.5 (± 24.5)	44.0 (± 35.4)	51.0 (± 32.2)

3) By mobility

	**Fiji**		**Indonesia**	
	Walking* n = 72	Other n = 16	Walking* n = 67	Other n = 17
	**Number of children (%)**		**Number of children (%)**	
Porch of own home	61 (85%)	10 (63%)	53 (79%)	16 (94%)
Porch of another house	18 (25%)	1 (6%)	46 (69%)	9 (53%)
Outside own home	51 (71%)	10 (63%)	47 (70%)	10 (59%)
Outside another house	36 (50%)	2 (13%)	47 (70%)	13 (77%)
Outside in communal area	38 (53%)	2 (13%)	64 (96%)	13 (77%)
	**Proportion of time in % Mean (± SD)**		**Proportion of time in % Mean (± SD)**	
Porch of own home	55.0 (± 42.6)	54.0 (± 49.7)	22.6 (± 28.7)	37.8 (± 39.7)
Porch of another house	4.1 (± 15.0)	6.2 (± 24.7)	7.9 (± 13.0)	13.7 (± 24.1)
Outside own home	17.6 (± 26.2)	32.4 (± 45.1)	10.7 (± 18.5)	13.4 (± 24.1)
Outside another house	7.6 (± 15.2)	6.2 (± 23.3)	5.8 (± 10.5)	8.0 (± 17.5)
Outside in communal area	15.7 (± 25.0)	1.3 (± 4.9)	53.1 (± 31.3)	27.1 (± 35.7)

*Walking refers to children who were only walking, not those who were walking and also crawling

4) By sex

	Fiji		Indonesia	
	Female<br>n = 37	Male<br>n = 51	Female<br>n = 40	Male<br>n = 44
	**Number of children (%)**		**Number of children (%)**	
Porch of own home	28 (76%)	43 (84%)	38 (95%)	31 (71%)
Porch of another house	5 (14%)	14 (28%)	26 (65%)	29 (66%)
Outside own home	26 (70%)	35 (69%)	27 (68%)	30 (68%)
Outside another house	12 (32%)	26 (51%)	30 (75%)	30 (68%)
Outside in communal area	13 (35%)	27 (53%)	37 (93%)	40 (91%)
	**Proportion of time in %<br>Mean (± SD)**		**Proportion of time in %<br>Mean (± SD)**	
Porch of own home	46.8 (± 45.6)	60.6 (± 41.6)	27.5 (± 29.7)	24 (± 33.4)
Porch of another house	4.6 (± 18.0)	4.4 (± 16.4)	11.8 (± 18.5)	6.5 (± 12.7)
Outside own home	27.9 (± 38.0)	14.7 (± 23.0)	9.8 (± 20.7)	12.6 (± 18.7)
Outside another house	8.7 (± 21.1)	6.4 (± 12.9)	4.2 (± 9.3)	8.1 (± 14.2)
Outside in communal area	12.0 (± 26.0)	13.9 (± 21.4)	46.8 (± 32.2)	48.8 (± 35.3)

Table S9. Summary of Pearson's Chi-squared test with Yates' continuity correction (age and sex for each country) for proportion of children engaged in each high-risk context.

High-risk context	Country	X^2	p-value
Around/interaction with feces	Fiji	*	*
	Indonesia	*	*
Around/interaction with animals	Fiji	0.04	0.837
	Indonesia	1.49	0.223
Interaction with water	Fiji	*	*
	Indonesia	1.46	0.228

*Results not shown for tests with ≥1 cell count <5, as Chi-squared assumptions were violated

Table S10. Number of children observed mouthing any object when they were outdoor vs indoor as a proportion of all children, number of children observed mouthing outdoor vs indoor in each context (as a proportion of children observed mouthing in each location), and number of children observed mouthing specific object-groups in each location-context setting (as a proportion of children observed mouthing in each location-context setting).

**Fiji**	**All children**			
	106			
**Location**	**Outdoor**		**Indoor**	
Number of children observed mouthing any object when outdoors and indoors (proportion out of all children)	80 (76%)		103 (97%)	
**Context**	**High-risk**	**Other**	**High-risk**	**Other**
Number of children observed mouthing any object when outdoor/indoor in a high-risk or other context (proportion out of children mouthing any object when outdoors/indoors)	30 (38%)	79 (99%)	17 (17%)	102 (99%)
Number of children observed mouthing each object groups (proportion out of children mouthing any object when outdoor/indoor in a high-risk or other context):				
Food	7 (23%)	50 (63%)	9 (53%)	97 (95%)
Fomite	17 (57%)	73 (92%)	14 (82%)	96 (94%)
Own hand	21 (70%)	75 (95%)	12 (71%)	99 (97%)
Other hand	7 (23%)	44 (56%)	5 (29%)	84 (82%)
High-risk objects	6 (20%)	27 (34%)	7 (41%)	39 (38%)

**Indonesia**	**All children**			
	86			
**Location**	**Outdoor**		**Indoor**	
Number of children observed mouthing any object when outdoors and indoors (proportion out of all children)	82 (95%)		84 (98%)	
**Context**	**High-risk**	**Other**	**High-risk**	**Other**
Number of children observed mouthing any object when outdoor/indoor in a high-risk or other context (proportion out of children mouthing any object when outdoors/indoors)	60 (73%)	82 (100%)	20 (24%)	84 (100%)
Number of children observed mouthing each object groups (proportion out of children mouthing any object when outdoor/indoor in a high-risk or other context):				
Food	39 (65%)	74 (90%)	14 (70%)	77 (92%)
Fomite	42 (70%)	77 (94%)	12 (60%)	80 (95%)
Own hand	45 (75%)	80 (98%)	15 (75%)	82 (98%)
Other hand	23 (38%)	70 (85%)	7 (35%)	64 (76%)
High-risk objects	8 (13%)	23 (28%)	2 (10%)	21 (25%)

Table S11. Median and interquartile range (IQR) of children all-objects mouthing frequency, mouthing frequency when outdoor vs indoor, mouthing frequency when outdoor vs indoor in a high-risk/other context, and mouthing frequency for each object-groups in each category of location and context.

**Fiji**	**All mouthing frequency (contacts/h observed) (n = 106)[a]**			
	68.2 (37.7)			
**Location**	**Outdoor (n = 88)[b]**		**Indoor (n = 105)[b]**	
	50.1 (37.3)		71.8 (38.1)	
**Context**	**High-risk n = 58[c]**	**Other n = 78[c]**	**High-risk n = 32[c]**	**Other n = 103[c]**
	3.4 (40.0)	44.6 (37.0)	26.7 (68.2)	74.7 (42.1)
Food	0.0 (0.0)	2.7 (15.8)	0.0 (2.3)	19.3 (22.5)
Fomite	0.0 (3.2)	13.8 (18.5)	0.0 (23.7)	24.5 (27.5)
Own hand	0.0 (15.2)	14.3 (18.9)	0.0 (12.1)	19.4 (21.5)
Other hand	0.0 (0.0)	0.7 (4.3)	0.0 (0.0)	1.8 (5.3)
High-risk objects	0.0 (0.0)	0.0 (1.2)	0.0 (0.0)	0.0 (0.5)

**Indonesia**	**All mouthing frequency (contacts/h observed) (n = 86)[a]**			
	68.6 (37.9)			
**Location**	**Outdoor (n = 84)[b]**		**Indoor (n = 85)[b]**	
	58.9 (56.4)		74.7 (45.3)	
**Context**	**High-risk n = 76[c]**	**Other n = 81[c]**	**High-risk n = 44[c]**	**Other n = 81[c]**
	45.1 (75.9)	60.1 (50.7)	0.0 (93.0)	78.7 (47.0)
Food	2.4 (31.2)	19.6 (24.5)	0.0 (16.1)	28.0 (31.5)
Fomite	3.3 (17.1)	12.8 (13.0)	0.0 (7.3)	22.9 (25.2)
Own hand	6.2 (20.9)	15.9 (17.2)	0.0 (8.8)	16.1 (17.0)
Other hand	0.0 (3.6)	2.7 (4.9)	0.0 (0.0)	2.6 (4.2)
High-risk objects	0.0 (0.0)	0.0 (0.3)	0.0 (0.0)	0.0 (0.1)

[a]Calculated by dividing all mouthing counts with the child's video observation time, n is children who were observed for each country

[b]Calculated by dividing mouthing counts when outdoor or indoor with the child's outdoor/indoor time, n is children who spent time outdoor/indoor

[c]Calculated by dividing mouthing counts for each specific location and context with the child's specific location-context time, n is children who spent time in each location-context
